# Temporal Evolution of Inflammation and Neurodegeneration With Alpha-Synuclein Propagation in Parkinson's Disease Mouse Model

**DOI:** 10.3389/fnint.2021.715190

**Published:** 2021-10-05

**Authors:** Thuy Thi Lai, Yun Joong Kim, Phuong Thi Nguyen, Young Ho Koh, Tinh Thi Nguyen, Hyeo-il Ma, Young Eun Kim

**Affiliations:** ^1^Department of Biomedical Gerontology, Graduate School of Hallym University, Chuncheon, South Korea; ^2^Department of Neurology, Hallym University Sacred Heart Hospital, Hallym University, Anyang, South Korea; ^3^Hallym Neurological Institute, Hallym University, Anyang, South Korea; ^4^Department of Neurology, Yongin Severance Hospital, Yonsei University College of Medicine, Yongin, South Korea; ^5^Ilsong Institute of Life Science, Hallym University, Anyang, South Korea

**Keywords:** alpha-synuclein, microglia, astrocytes, neurodegeneration, Parkinson's disease

## Abstract

According to a few studies, α-synuclein (αSyn) propagation has been suggested to play a key role in the pathomechanism of Parkinson's disease (PD), but neurodegeneration and the involvement of inflammation in its pathologic progression are not well understood with regard to temporal relationship. In this study, with the help of the PD mouse model injected with intrastriatal αSyn preformed fibril (PFF), the temporal evolution of αSyn propagation, inflammation, and neurodegeneration was explored in the perspective of the striatum and the whole brain. In the PFF-injected striatum, inflammatory response cells, including microglia and astrocytes, were activated at the earliest stage and reduced with time, and the phosphorylated form of αSyn accumulation increased behind it. Afterward, the degeneration of striatal dopaminergic neurons became significant with the conspicuity of behavioral phenotype. Similar patterns of forefront eruption of inflammation and then followed by αSyn propagation were noted in the opposite striatum, which were not injured by PFF injection. In analyzing the whole brain, inflammatory responses were activated at the earliest stage, and the soluble αSyn expression increased concurrently. The inflammatory response decreased afterward, and the accumulation of the insoluble form of αSyn increased behind it. Our results suggested that the inflammatory response may precede the accumulation of the pathologic form of αSyn; thereafter, the neurodegeneration and motor dysfunction followed αSyn proliferation in the PD mouse model. From this model, recognizing the temporal relationship between inflammation, αSyn propagation, and neurodegeneration may be helpful in establishing the PD animal model and monitoring the effect of interventional therapy.

## Introduction

Parkinson's disease (PD), which has been considered as the second most common neurodegenerative disease, is characterized by a profound loss of nigrostriatal dopaminergic neurons and its relevant clinical features of Parkinsonism—bradykinesia, rigidity, gait disturbance, and tremor (Kouli et al., 2018). Additionally, the accumulation and spread of alpha-synuclein (αSyn) protein have been suggested as the main disease mechanisms, and its consequent fibrillar intracellular inclusion—Lewy bodies and Lewy neurites—is regarded as the pathologic hallmark of PD (Nuber et al., 2013; Kouli et al., 2018). However, the relationship between this αSyn deposition, neuronal loss, and other arresting mechanisms during the initiation and progression of PD remains to be undetermined (Samii et al., 2004).

In addition to αSyn propagation, neuroinflammation has been suggested as one of the main mechanisms contributing to PD pathogenesis (Harms et al., 2020; Hirsch and Standaert, 2020). Postmortem studies using the PD brain reported extensive microgliosis in the brain regions affected by synuclein pathology (Brás et al., 2020; Harms et al., 2020). In addition to microglia, astrocytic activation, and increased MHC-II, MHC-I expression has also been reported to contribute to the progression of PD (Choi et al., 2020; Rostami et al., 2020). In another aspect, the neuroprotective function of microglia in the αSyn transmission was proposed (Xia et al., 2019). Although the precise role of inflammation in this αSyn-associated disease process is not fully understood, inflammation may be not just the result of the disease process but may be the contributor in the progressive nature of synucleinopathy (Xia et al., 2019; Brás et al., 2020; Guo et al., 2020; Harms et al., 2020; Hirsch and Standaert, 2020).

In recent years, artificially made αSyn preformed fibril- (PFF-) injected animal model has been noted to mimic αSyn propagation hypothesis, and this model may be valuable in studying αSyn spreading, neuroinflammation, and neurodegeneration (Chung et al., 2019). Taking advantage of this animal model, the evaluation and standardization of the temporal change of inflammatory response, the development of neurodegeneration, and behavioral phenotype manifestation during αSyn propagation were planned. This study suggests that the inflammatory response, especially microglia, may precede the accumulation and propagation of αSyn after PFF injection; thereafter, neurodegeneration and parkinsonian clinical features were noted in the PD mouse model following αSyn proliferation.

## Materials and Methods

### Mouse Husbandry

Young adult wild-type mice (C57BL/6N, male) aged 2.5 months with a weight average of 23.5 g (Young Bio Company, Seoul, Korea) were used in this study. All animals were housed at a constant room temperature (RT) with a 12-h light–dark cycle in the animal facility of Ilsong Institute of Life Science, Hallym University, Korea. New bedding and food were replaced every week until sacrifice. The Animal Research Committee of Hallym University Sacred Heart Hospital granted ethical approval and permission to this study (HMC2019-1-1130-42). All methods were performed in accordance with the relevant guidelines and regulations in the Section “Methods”.

### Production of Recombinant αSyn Monomer and the Generation of αSyn PFF

PD434-SR mouse αSyn vector was purchased from Addgene (Watertown, MA, USA, plasmid #89073, https://www.addgene.org/89073). Mouse αSyn vector was then amplified in *Escherichia coli* DH5α; later on, the expression vector was transformed into *E. coli* BL-21 bacteria. A single colony was selected for growth in 2xYT medium supplemented with 100 μg/ml ampicillin. Growth was monitored to log phase until the bacterial density of OD600 reached 0.3–0.6, which was followed by protein induction using 0.5 mM IPTG for 5 h at 37°C on a shaker at 100 rpm. Harvest cells were then performed by centrifugation at 3,000 rpm for 15 min at 4°C. Pellet was then collected, and ~5 g of cells were resuspended in 5 ml distilled water, which were supplemented with 50 μl of saturated MgCl_2_. Next, cell solutions were aliquoted into 1.5-ml Eppendorf tubes (Hamburg, Germany), and were kept at −80°C overnight. Afterward, homogenates were boiled at 98°C for 30 min and were centrifuged at 13,000 rpm for 20 min. The supernatant was then collected, and further dialysis was performed. Briefly, αSyn protein sample was placed into 2 L ice-cold 20 mM Tris-HCl with a pH of 8.0 buffer overnight. Next, the solution was filtered using a 0.2-μm membrane to discard the aggregated protein; then the purification step was ion exchange. The protein was purified using the HiTrap column (GE Healthcare, Chicago, IL, USA) with buffer A containing 20 mM Tris-HCl, with a pH of 8.0, and buffer B containing 20 mM Tris-HCl and 1 M NaCl, with a pH of 8.0. Eluted fraction containing αSyn was collected and was further dialyzed in water at 4°C overnight. Dialyzed protein sample was then frozen until completely dry, and αSyn protein was resolved in 20 mM Tris-HCl and 150 mM NaCl, with a pH of 8.0. Finally, the protein sample was centrifuged at 13,000 rpm at 4°C, and the supernatant containing αSyn was collected and was kept at −80°C until investigation. Although the level of endotoxin was low, we compared the inflammation by monomeric αSyn with PFF to exclude the endotoxin effect.

For the production of αSyn PFF, a siliconized 1.5-ml Eppendorf tube (Hamburg, Germany) was prepared to prevent the surface absorption of protein using Sigmacote. Next, 1 ml of monomeric αSyn was added to the tube coupled with 10 μl 5% sodium azide and two 5-mm glass beads. The tube was shaken at 800 rpm at 37°C for 10 days on the Thermoshaker. PFF was collected, and protein concentration was measured.

### Production of the PD Mouse Model

The mouse αSyn PFF (2.5 mg/ml) prepared for this study was stored at −80°C before use. PFF was taken out from the freezer and was put on ice. A well-constructed αSyn PFF was viewed using a transmission electron microscope, and a Thioflavin T assay was performed for the control of PFF quality. The obtained PFF was then sonicated for 10 min at 37°C in an ultrasonic water bath before its use (Supplementary Figures 1A,B).

**Figure 1:**
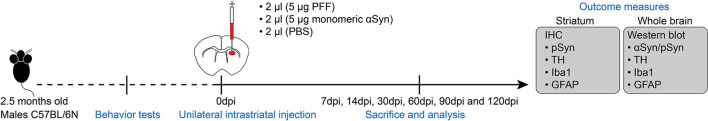
Experimental schematic. C57BL/6 male mice aged at 2.5 months old were received preformed fibril (PFF) or control substances [monomeric alpha-synuclein (αSyn) or phosphate buffered saline (PBS)] into the unilateral dorsal striatum, and behavioral tests were done at baseline and before sacrifice. The mouse brain was isolated at each time point for analysis using immunohistochemistry and a western blot as provided in detail. dpi, day postinjection.

Animals aged 2.5 months of age were anesthetized *via* intraperitoneal (i.p.) injection of avertin solution (240 mg/kg, 0.2 ml/10 g), and ketoprofen (0.2 cc of 1%, ketoprofen, s.c.) was injected to relieve pain. The unilateral intracranial injection was performed into the right striatum with 2 μl recombinant αSyn PFF [the final concentration of PFF is 5 μg/2 μl, using a 10-μl Hamilton syringe (no. 2)], with a speed of 1 μl/min using a stereotaxic instrument. Control animals were administered with 5-μg/2-μl monomeric αSyn or 2-μl sterile phosphate buffered saline (PBS) into the right striatum using the same method with PFF (Figure 1). The right dorsal striatum was then targeted using the following coordinates from the bregma: anterior-posterior +0.5 mm, mediolateral −2 mm, and dorsoventral in the depth of −3 mm according to the mouse brain atlas. After the injection, the needle was kept for 5 min to fix the solution at the position; then the syringe was removed slowly by turning upward to −1.5 mm (half of the depth) and was kept for 1 min, and the syringe was removed out of the brain. After surgery, animals were taken care of using i.p. saline and subcutaneous ketoprofen injection for 3 consecutive days postinjection (dpi). The mouse was sacrificed for an analysis at baseline without PFF injection (0 day), at 7 days after PFF injection, i.e., 7, 14, 30, 60, 90, and 120 dpi. Control animals consisted of PBS-injected mice, which were sacrificed at 7, 30, 60, and 90 dpi, and monomeric αSyn-injected mice, which were sacrificed at 7 and 120 dpi (Figure 1).

### Sacrifice and Tissue Preparation

Before sacrificing, the animals were anesthetized using avertin solution (240 mg/kg, 0.2 ml/10 g, i.p.) and were transcardially perfused with 50 ml of ice-cold 1× PBS, followed by 30 ml of ice-cold 4% paraformaldehyde (4% PFA). The brain was then taken out and was fixed in 4% PFA at 4°C overnight. The brains were then sectioned using vibratome at 40-μm thickness, and the cutting speed was 0.055 mm/s. Sections were collected from the olfactory to the substantia nigra (SN), and the sectioned tissues were put in order in a 96-well culture plate. They were stored in 0.05% sodium azide (0.05% NaN3) at 4°C until they were used for immunostaining.

### Immunofluorescence

Free-floating sections were soaked in 1x PBS in a12-well culture plate for 20 min at RT on a shaker before blocking by 0.5% Triton X-100 in PBS with a supplement of normal donkey serum (1:1,000) for 1 h on a shaker at RT. Brain sections were then incubated using the primary antibodies (Supplementary Table 1), which were diluted in 0.5% Triton X-100 in PBS solution overnight at 4°C. Then, the brain tissues were washed three times for 10 min in PBS at RT and then were incubated with secondary antibodies conjugated to Alexa Fluor 488, Alexa Fluor 555, DyLight 488, and DyLight 550 for 3 h. The experimental plate was covered with an aluminum foil at RT on the shaker to protect fluorescence. Sections were then washed three times for 10 min in PBS and were later transferred to the coated slide. Then, the slides were mounted with 4′-6-diamidino-2-phenylindole (DAPI) solution using Vectashield (Vector Laboratories, Burlingame, CA, USA) to visualize the cell nuclei; they were then stored in a dark box at 4°C.

### Immunohistochemistry

For immunohistochemical staining with anti-tyrosine hydroxylase (TH), anti-ionized calcium-binding adaptor molecule 1 (Iba1), anti-glial fibrillary acidic protein (GFAP), and anti-αSyn phospho-Ser129 primary antibodies (pSyn), the sections were washed in 1x PBS for 20 min on a shaker; then peroxidase endogenous blocking was applied using 0.3% H_2_O_2_ at RT for 15 min. For blocking the nonspecific binding of immunoglobulin, the sections were continuously incubated in 2% BSA in 0.3% Triton X/PBS for 1 h at RT. Primary antibodies were incubated overnight at 4°C and were further incubated with horseradish peroxidase- (HRP-) conjugated secondary antibodies for 2 h at RT. Next, the brain sections were washed three times using PBS for 10 min. 3,3′-diaminobenzidine (DAB) reagent was then prepared with the following compositions: two drops of buffer, four drops of DAB, and two drops of peroxidase oxide in 5 ml distilled water. The brain sections in a 1-ml DAB reagent for 3 min were then developed and were then immediately transferred to distilled water. Finally, the tissue was transferred to the coated slide with the mounting solution and was stored at 4°C.

### Imaging and Quantification

Fluorescence images were obtained using a confocal microscopy system (LSM 700, Carl Zeiss, Oberkochen, Germany) or Olympus BX51 conventional fluorescence microscopy with U-RFL-T power supply equipped with a 1.25x/0.04NA, 4x/0.1NA, 10X/0.3NA, 20X/0.5NA, and 40X/0.75NA objective lens. Fluorescence images of different brain regions, including the striatum, SN, piriform cortex, somatosensory cortex, amygdala, and hippocampus, were taken as shown in Supplementary Figure 1C. Images were processed using the Zen, Carl Zeiss, and ImageJ Fiji software.

To evaluate TH, GFAP-positive astrocyte, and Iba1-positive microglia immunoactivity in the whole mouse striatum and TH immunoactivity in the SN, coronal brain section images were obtained using an Olympus BX51 microscope equipped with a PLN 4X/0.1NA objective lens, and all parameters in terms of exposure time, contrast, and resolution were set in a similar manner. A semiquantitative optical density (OD) analysis of DAB images was carried out using the ImageJ Fiji software as per the modified protocol previously described (Tagliaferro et al., 2006; Stefanova et al., 2012; Crowe and Yue, 2019). Briefly, the color of all images was corrected by applying color deconvolution with the DAB vector function in ImageJ. Next, the striatum of four 40-μm slices from each independent mouse was measured, one in each PFF-injected striatum and one in noninjected striatum at each time point by drawing the region of interest from the four sections per brain region to yield eight mean gray values with the values of 0 and 255 for black and white, respectively. The observed gray level was converted to relative OD using the following formula OD = log (255/mean gray level) (Tagliaferro et al., 2006; Stefanova et al., 2012). Therefore, eight OD values in each animal were obtained. The measurement was then replicated in each time point. The resulting average OD in each time point was represented as the measurement for TH degeneration and microglial activation or astrocytic activation.

To evaluate pSyn OD, a similar method as mentioned earlier was applied on DAB images using the ImageJ Fiji software. Briefly, pSyn OD measurement was carried out on the images that were captured at 10X magnification using a PLN 10X/0.3NA objective lens, and all parameters in terms of exposure time, contrast, and resolution were the same. The same method of OD was also applied to estimate pSyn expression in the striatum. The OD value in each animal and the measurement were replicated at each time point. The resulting average OD value was represented by pSyn OD in each mouse.

### Western Blot

The biochemical analysis of the mouse brain was performed as per the modified protocol previously described (Yun et al., 2018). Briefly, the mouse brain was dissected into two parts, i.e., the PFF-injected side and noninjected side of the brain excluding the cerebellum. The brain was homogenized in a soluble nonionic detergent (NP40-soluble fraction) and an ionic detergent (NP40-insoluble fraction). First, the mouse brain was homogenized in a nonionic detergent, which contained 10 mM Tris-HCl, pH 7.4, 150 mM NaCl, 5 mM ethylenediaminetetraacetic acid (EDTA), 0.5% Nonidet P-40, 1 × phosSTOP, and 1 × protease inhibitor cocktail. The homogenate was centrifuged at 12,000 rpm at 4°C for 20 min (centrifuge 5430R). Then, the resulting pellet and supernatant part (NP40-soluble fraction) were collected. The pellet was washed two times in a nonionic lysis buffer and was further homogenized in the ionic detergent, which contained 10 mM Tris-HCl, pH 7.4, 150 mM NaCl, 5 mM EDTA, 0.5% Nonidet P-40, 1% sodium dodecyl sulfate (SDS), 0.5% sodium deoxycholate, 1 × phosSTOP, and 1 × protease inhibitor cocktail. Then, the homogenate was incubated on ice for 5 min and was further centrifuged at 12,000 rpm at 4°C for 40 min. Then, the resulting supernatant (NP40-insoluble fraction) was collected. Protein concentrations were determined using the bicinchoninic acid (BCA) assay. Proteins were kept in aliquots at −80°C until investigation.

The protein samples were treated for 3 min at 100°C in the sample-treated dye solution (5× Laemmli sample buffer) containing 2% SDS and 5% β-mercaptoethanol. Equal amounts of the protein samples from each group in a soluble or an insoluble fraction were subjected to 12% SDS polyacrylamide gel electrophoresis (PAGE) for 2 h at 80 V. The gel was transferred to a methanol-activated poly vinylidene fluoride (PVDF) membrane (exposed in methanol 100% for 1 min and soaked in transfer buffer before use) (Immobilon-P, pore size 0.45 μm, and cut size 6 cm × 8 cm) to detect the interested proteins using the Mini Trans-Blot electrophoretic transfer cell (Bio-Rad, Hercules, CA, USA) at 250 mA 90 V constant current for 1 h and 30 min using a Model 200/2.0 Power Supply (Bio-Rad, Hercules, CA, USA). Next, the transferred membranes were soaked in Ponceau S solution to be able to visualize the proteins and were washed three times for 5 min in 1x-TBST buffer.

For αSyn detection, the transferred membranes were treated using 0.4% PFA in PBS for 30 min at RT as previously described (Sasaki et al., 2015) to increase detection sensitivity. After the incubation, the membrane was washed three times in 1x-TBST for 5 min and in 5% (w/v) nonfat dry milk blocking for 1 h at RT. The membranes were then incubated with primary antibodies (ab212184, ab51253, Abcam, Cambridge, UK) in a blocking buffer at 4°C overnight and were washed three times for 5 min in 1x-TBST buffer, which was followed by an incubation with secondary antibodies (HRP conjugate) in a blocking buffer for 1 h at RT.

For a capable detection of the presence of other proteins, the transferred membranes were blocked in 5% (w/v) nonfat dry milk for 1 h at RT. The membranes were then washed three times in 1x-TBST for 5 min, and they were then incubated in primary antibodies at 4°C overnight. Next, the membranes were washed three times for 5 min in 1x-TBST buffer and were then incubated in secondary antibodies (HRP conjugate) for 1 h at RT. Finally, protein detections were conducted using the electrochemiluminescence (ECL) reagent and then were detected using the ChemiDoc MP Imaging System. Quantitative comparisons between the samples on the blots were processed in parallel. All raw blot and gel images are available in Supplementary Figure 7.

### Behavioral Testing

To assess the deterioration of motor symptoms, behavioral tests, including rotarod, wire hang, and clasping tests, were conducted at two time points—baseline (before injection at 2.5 months old) and at sacrifice day.

### Rotarod Test

The rotarod test was performed following the previous report with some modifications (Luk et al., 2012a). Briefly, mice run on a 2.5-cm-diameter rotating apparatus at a speed of 40 rpm, and four initial training sessions composed of 5-min run and 5-min break were conducted. The rotarod machine was then cleaned with 70% ethanol in between each mouse. After an hour, the test trials were conducted, and the time each animal remained on the rotarod was recorded. Each mouse was able to stay on the rotarod for at least 30 s, and animals not falling off the rotarod after 300 s were given a maximum score of 300 s (≤30 s *x* ≤300 s). Each mouse was tested three times, and the average time was used for statistical analysis.

### Wire Hang Test

The wire hang test was also performed (Luk et al., 2012a). To summarize this, the mice were hanged on a 55-cm-long 2-mm-thick wire at a height of 90 cm. Four initial training sessions composed of 5-min hang and 5-min break were conducted. After an hour, the test trials were conducted, and the latency of mice to fall off the wire was recorded. Each mouse should stay on the wire for at least 10 s, and animals not falling off after 300 s were given a maximum score of 300 s (≤10 s *x* ≤300 s). The average values from the three trials were then used for analysis.

### Clasping Test

Hind limb clasping during tail suspension was recorded to assess the behavioral phenotype and disease progression during the αSyn propagation. It was recorded for 10 s for each mouse using a hand camera, and the score was measured as described in the following (Miedel et al., 2017). The experiment was not blinded but each test was performed multiple times and was done by video recording to ensure that the score is reproducible and to prevent bias. The score of hind limb clasping was assigned from 0 to 4: 0, no limb clasping, normal escape extension; 1, one hind limb exhibits incomplete splay with toes exhibiting normal splay; 2, both hind limbs exhibit incomplete splay with toes exhibiting normal splay; 3, both hind limbs exhibit clasping with curled toes and immobility; and 4, forelimbs and hind limbs exhibit clasping and are crossed with curled toes and immobility (Miedel et al., 2017).

### Statistical Analysis

In this study, the statistical analysis of data was performed using Microsoft Excel and GraphPad Prism version 5 software (https://www.graphpad.com/). The mean value of OD in different groups and the score of the clasping test between groups were compared using nonparametric ANOVA (the Kruskal–Wallis test), which were then followed by a *post hoc* analysis (Dunn's test). Densitometric analysis of a western blot was performed using the Fiji (ImageJ) software, and the final values were compared using nonparametric ANOVA (the Kruskal–Wallis test), which was followed by a *post hoc* analysis and significance means the comparison of each time point to baseline (0 dpi). The comparison of rotarod and wire hang test results at baseline and each time postinjection were performed using the Wilcoxon signed-rank test. The results were considered to be statistically significant for ^*^p < 0.05, ^**^p < 0.01, and ^***^p < 0.001.

## Results

### Spreading of Pathological αSyn Into Various Brain Regions by αSyn PFF Injection

The longitudinal pattern of αSyn aggregation and spreading in various brain regions, including striatum, SN, somatosensory cortex, piriform cortex, amygdala, and hippocampus, were evaluated at different time points, including at baseline (0 dpi without any injection), 7, 14, 30, 60, 90, and 120 dpi (Figure 1 and Supplementary Figures 1C, 2).

**Figure 2:**
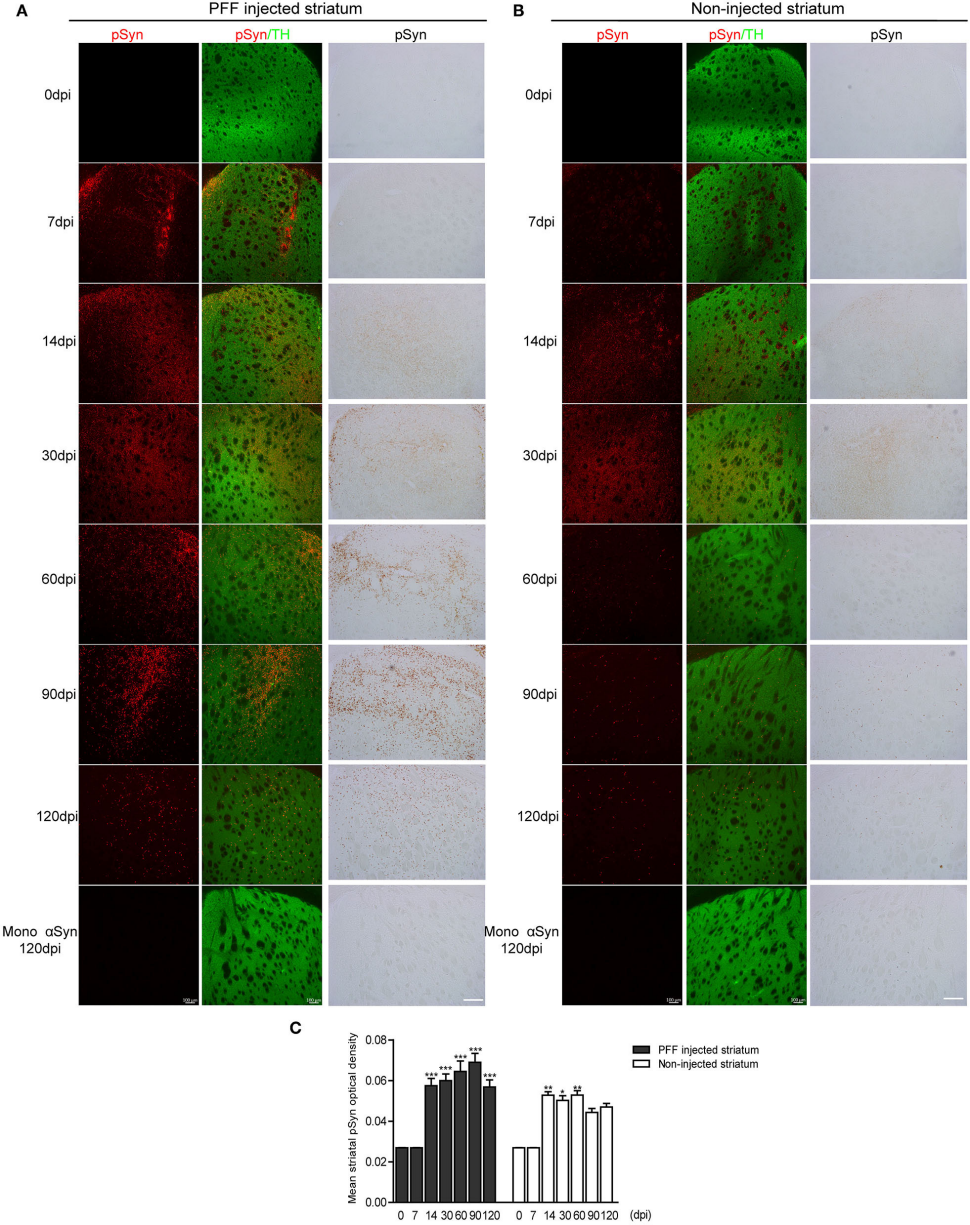
The temporal accumulation of pathological αSyn in the striatum following unilateral striatal PFF injection. **(A,B)** Representative images of pSyn inclusions in the striatum at different time points. Confocal images showing double immunostaining of pSyn (red) and tyrosine hydroxylase (TH) (green) (left column), and 3,3′-diaminobenzidine (DAB) staining images of pSyn (right column) in the PFF or control- (monomeric αSyn) injected striatum **(A)** and noninjected striatum **(B)**. **(C)** The mean striatal pSyn optical density (OD) using DAB images was quantified (χ^2^ = 92.84, p < 0.0001, df = 13). Bars indicate SEM, n = 3–4/time point. Significance means the comparison of each time point to baseline (0 dpi) using *post hoc* analysis (the Kruskal–Wallis test, ^*^p < 0.05, ^**^p < 0.01, ^***^p < 0.001). Scale bar, 100 μm.

Firstly, the pathological αSyn propagation in the striatum was evaluated because PFF was injected in this region and statistical analysis was measured using the comparison of baseline (0 dpi) and each time point. Using immunostaining, pSyn-positive inclusions in the bilateral striatum were noted from 7 dpi (Figures 2A,B). pSyn-positive inclusions were observed to increase with time until 90 dpi and then tended to decrease at 120 dpi in the PFF-injected striatum (Figures 2A,C). In the noninjected striatum, pSyn inclusions were increased and plateaued from 14 to 60 dpi and tended to decrease from the maximum value (Figures 2B,C). No αSyn aggregations were found in the mouse brain at 0 dpi and in the monomeric αSyn-injected mice at 120 dpi (Figures 2A,B).

**Figure 3:**
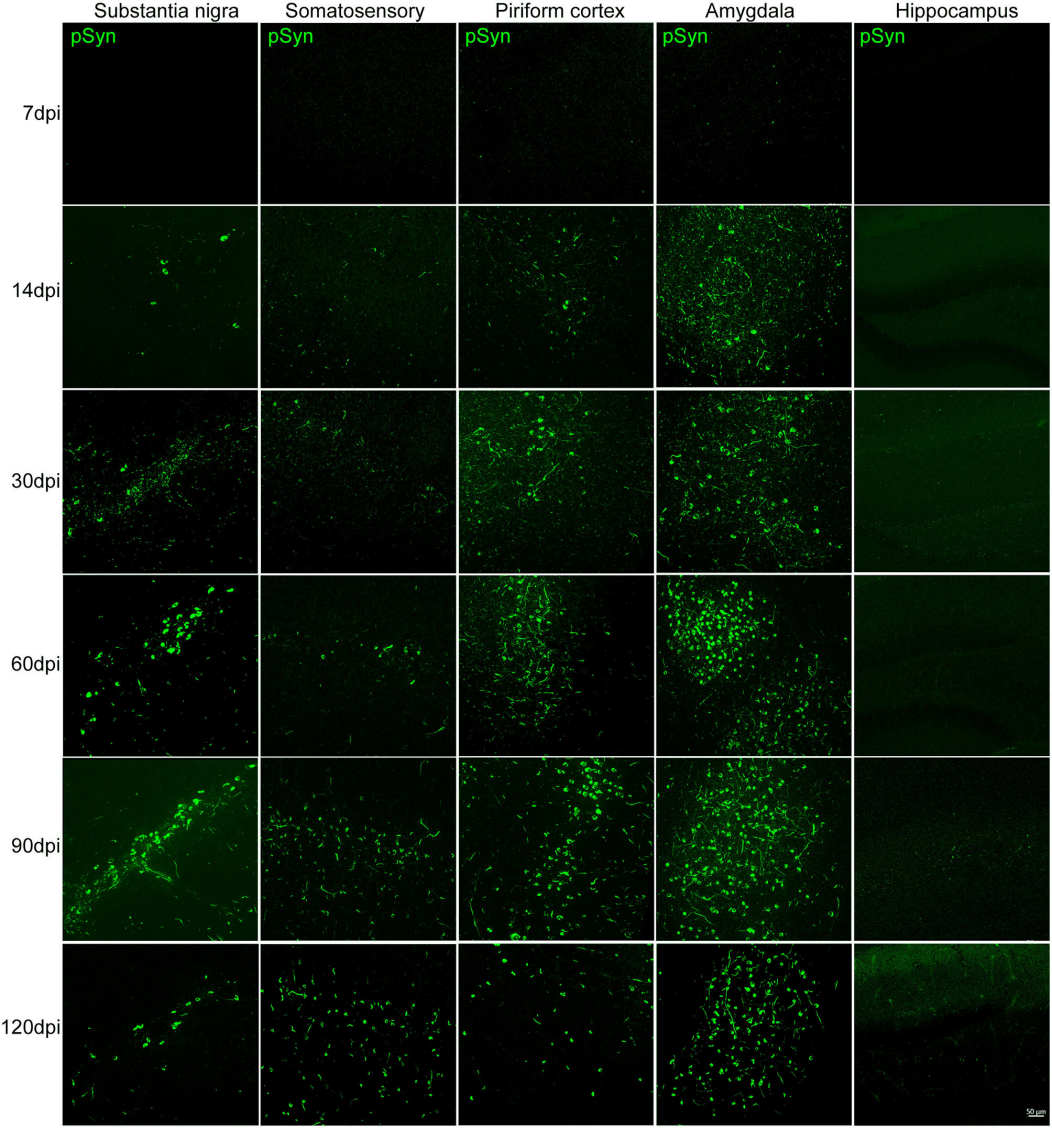
The accumulation of pathological αSyn in various brain regions at different time points. Representative images of αSyn inclusions in different brain areas at each time point in the PFF-injected side. Immunofluorescence analysis of αSyn (green) was performed in the substantia nigra (SN), somatosensory cortex, piriform cortex, amygdala, and hippocampus of PFF-injected mice at different inoculation time. Scale bar, 50 μm.

The presence of increasing αSyn inclusions in the SN was detected with time (Figure 3 and Supplementary Figure 2A). The pattern of pathological αSyn accumulation in various brain regions beyond the nigrostriatal areas was also detected at each time point. The abundance of pSyn-positive accumulation was observed in various brain regions, including the somatosensory cortex, piriform cortex, amygdala, and hippocampus, over time as shown in Figure 3 and Supplementary Figures 2B–D.

**Figure 4:**
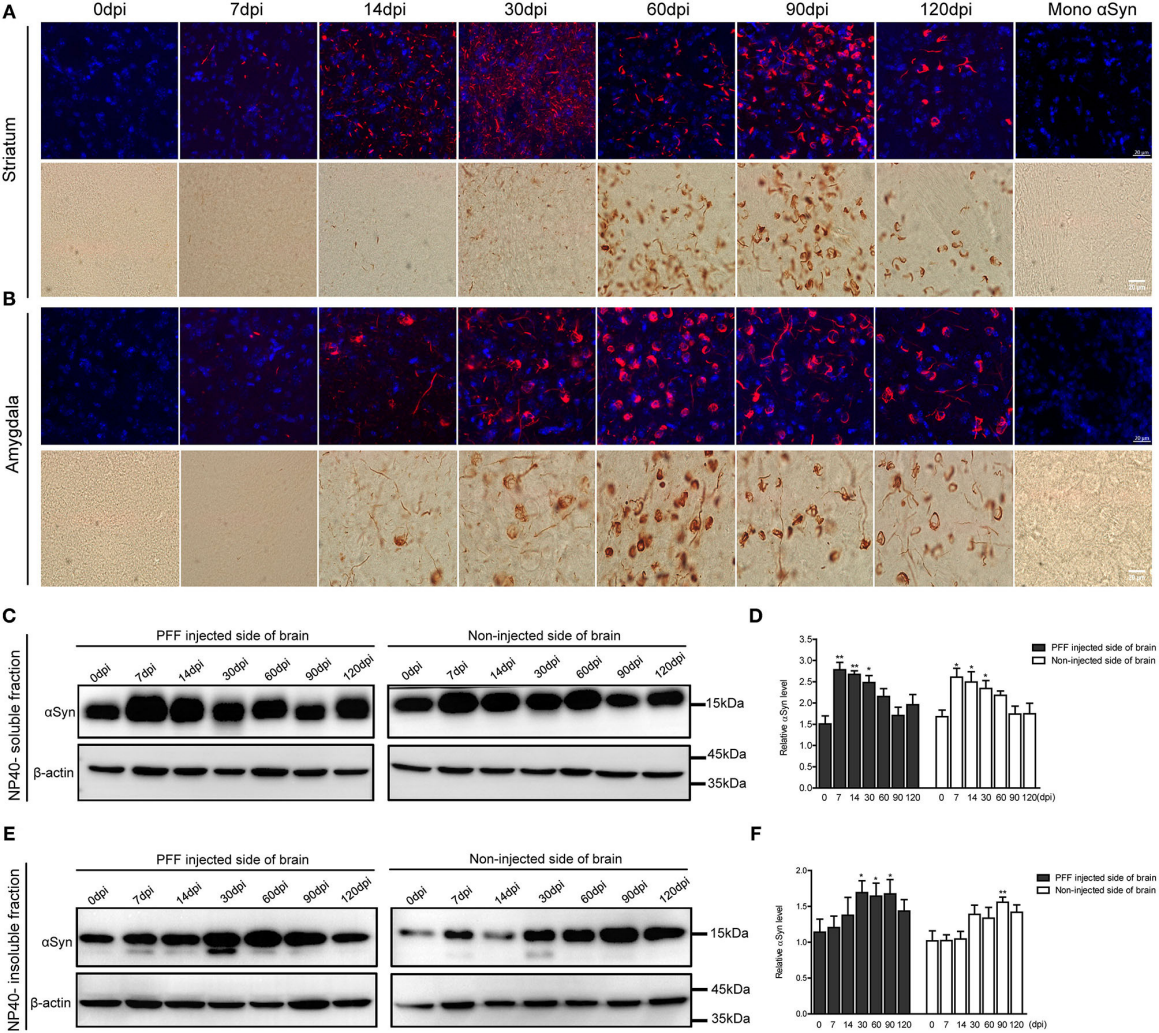
Morphological changes of αSyn accumulation and the temporal change of αSyn contents in the brain of PFF-injected mice. **(A,B)** Phosphorylated αSyn positive inclusions changed their structures with time in the brain including striatum **(A)** and amygdala **(B)**. Upper images showing the confocal images of pSyn (red) and 4′ 6-diamidino-2-phenylindole (DAPI) (blue) at different time points. Bottom images showing the detection of pSyn structures using DAB staining at each time point. Scale bar, 20 μm. **(C–F)** αSyn contents (antibody to αSyn monomer, ab212184) in the PFF-injected side of the whole brain and the noninjected side of the whole brain were evaluated in NP-40 soluble fraction **(C,D)** and insoluble fraction **(E,F)**. **(C,D)** Soluble αSyn level showed a significant increment at an early time point and then decreased with time in a western blot **(C)** and its quantification **(D)** (χ^2^ = 40.66, *p* = 0.0001, *df* = 13). **(E,F)** αSyn level in NP-40 insoluble fraction increased in PFF-injected and noninjected sides (χ^2^ = 27.44, *p* = 0.0108, *df* = 13). Original blots are presented in Supplementary Figure 7. Bars indicate SEM, *n* = 4–5/time point. Statistical analysis was measured with the comparison of 0 dpi and each time point by *post hoc* analysis (the Kruskal–Wallis test, ^*^p < 0.05, ^**^p < 0.01).

The morphological alteration of aggregated αSyn inclusions with time was observed in various brain regions with a similar pattern. Grossly, small dot-like or thread-like inclusion was observed at an early stage after PFF injection, and these structures have become more condensed and bigger and finally formed a Lewy body-like round structure over time (Figures 4A,B). Meanwhile, those morphological changes, i.e., the maturation of αSyn aggregation, showed to be faster in the amygdala than in the striatum or other brain regions (Figures 3, 4).

The change of αSyn protein content was further analyzed at different time points after the PFF injection from the whole brain. αSyn protein content was divided into NP40-soluble and NP40-insoluble fractions using the PFF-injected and noninjected side of each brain (Figures 4C–F). αSyn contents in the soluble fraction increased and peaked at 7 dpi and tended to decrease with time in both sides of the brain compared to baseline (Figures 4C,D). On the other hand, αSyn content in the insoluble fraction increased slowly compared to αSyn in soluble fraction in the brain (Figures 4E,F). The phosphorylated form of αSyn in an insoluble fraction showed the same trend with αSyn in an insoluble fraction as shown in Supplementary Figure 3.

### Dopaminergic Neurodegeneration During Pathological αSyn Transmission

**Figure 5:**
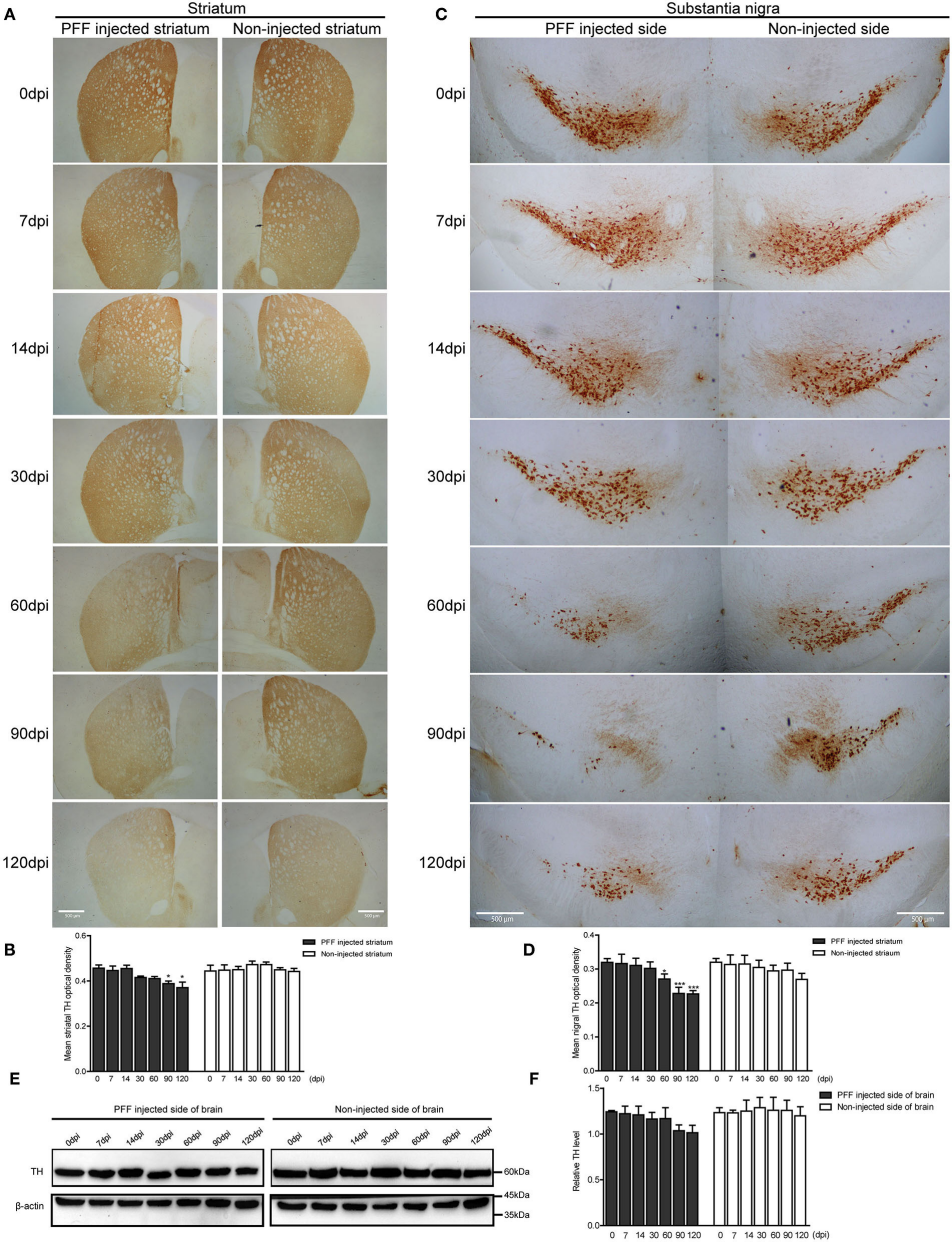
Dopaminergic neuronal density decreased in the striatum and SN following PFF injection. **(A,B)** TH-positive dopaminergic nerve terminal density in the striatum decreased with time in PFF-injected and noninjected sides, which are shown in the DAB image **(A)** and its quantification data **(B)** (χ^2^ = 41.72, p < 0.0001, df = 13). **(C,D)** TH-positive dopaminergic neurons decreased with time in the bilateral SN, especially PFF-injected SN, which is shown in the DAB image **(C)** and quantification **(D)** (χ^2^ = 33.64, p = 0.0014, df = 13). **(E)** The western blotting analysis of the whole brain lysate of the PFF-injected side of the brain and the noninjected side of the brain at various time points, using antibodies against TH. Original blots are presented in Supplementary Figure 7. **(F)** The relative TH expression level in the PFF-injected side of the brain (χ^2^ = 12.00, p = 0.5278, df = 13). Bars indicate SEM, n = 3–4/time point. Significance was measured by a comparison of 0 dpi to each time point by *post hoc* analysis (the Kruskal–Wallis test, ^*^p < 0.05, ^***^p < 0.001). Scale bar, 500 μm.

Dopaminergic nerve terminal density using TH immunoreactivity decreased in the PFF-injected striatum with time (Figure 5A). Image quantification showed that TH immunoreactivity decreased significantly at 90 and 120 dpi compared to baseline in the PFF-injected striatum while TH signal was not significantly decreased in the noninjected striatum during the observation period (Figure 5B).

In addition, the degeneration of TH-positive dopaminergic neurons was observed in SN. TH immunoreactivity in SN was significantly reduced in the PFF-injected side at 60, 90, and 120 dpi compared to 0 dpi, whereas the opposite side of SN showed the decreased tendency of TH signal without statistical significance (Figures 5C,D). For further investigation of dopaminergic neurodegeneration in the whole brain, a western blot was performed using the protein extracts from the PFF-injected and noninjected side of the whole brain (Figures 5E,F). The relative TH expression level tended to decrease in the PFF-injected side of the brain at 90 and 120 dpi, but it was not statistically significant yet compared to 0 dpi (Figure 5F).

### Microglial and Astrocytic Activation During αSyn Propagation

Inflammatory responses of the two main inflammatory cells—astrocytes and microglia—were observed during PFF-induced αSyn propagation. Immunohistochemistry was performed to measure the inflammatory response in the striatum, and a western blot was performed to evaluate the inflammation from the whole brain at each time point.

**Figure 6:**
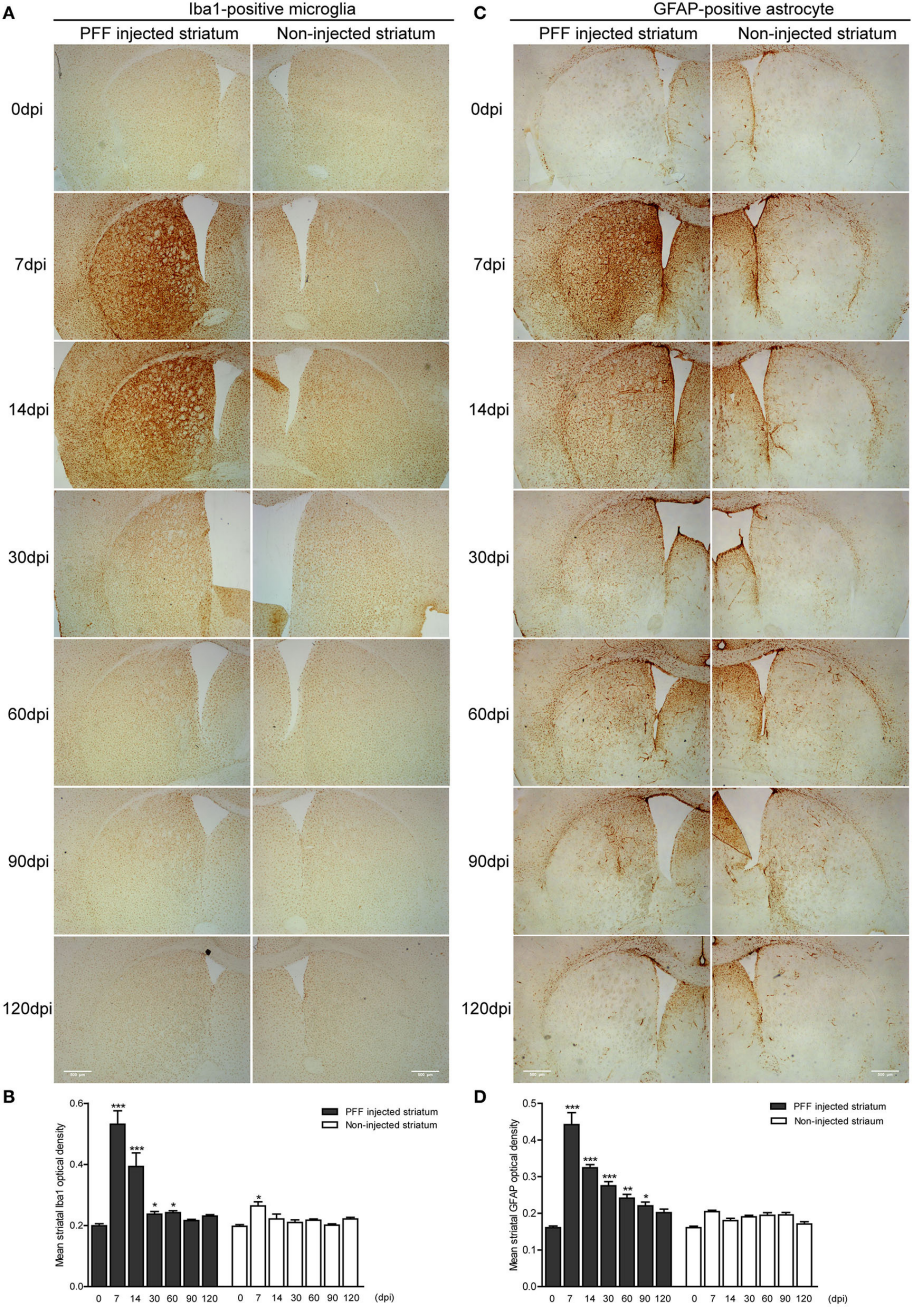
The temporal change of microglial and astrocytic activation in the striatum. **(A,B)** DAB images **(A)** and its OD **(B)** showed that ionized calcium-binding adaptor molecule 1- (Iba1-) positive microglial expression increased at 7 dpi and decreased with time in the PFF-injected (left column) and noninjected striatums (right column) (χ^2^ = 71.86, p < 0.0001, df = 13). **(C,D)** DAB images **(C)** and its OD (D) showed that glial fibrillary acidic protein- (GFAP-) positive astrocytic expression in PFF injected increased at 7 dpi and decreased with time (χ^2^ = 94.29, p < 0.0001, df = 13). Bars indicate SEM, n = 3–4/time point. Statistical significance was measured with a comparison of 0 dpi and each time point by *post hoc* analysis (the Kruskal–Wallis test, ^*^p < 0.05, ^**^p < 0.01, ^***^p < 0.001). Scale bar, 500 μm.

The abundance of microglial activation was observed in the bilateral striatum of PFF-injected mice (Figures 6A,B and Supplementary Figure 4A) while the striatum of PBS-injected mice or monomeric αSyn-injected mouse showed only a slight increase of microglia at 7 dpi (Supplementary Figures 4B,C, 5A). Microglial activation in the bilateral striatum of PFF-injected mice peaked at 7 dpi and was observed to decrease with time (Figures 6A,B).

The GFAP-stained astrocytic activation showed a similar temporal pattern with microglial activation in the striatum of the PFF-injected mouse (Figure 6C and Supplementary Figure 4D) while the striatum of PBS-injected mouse or monomeric αSyn-injected mouse showed a slight increase of astrocytes along the needle tract (Supplementary Figures 4E,F, 5B). A significant increase in GFAP-positive immunoreactivity was also observed in the PFF-injected striatum at 7 dpi on the PFF-injected side even though it was not statistically significant in the noninjected striatum compared to baseline (Figures 6C,D).

**Figure 7:**
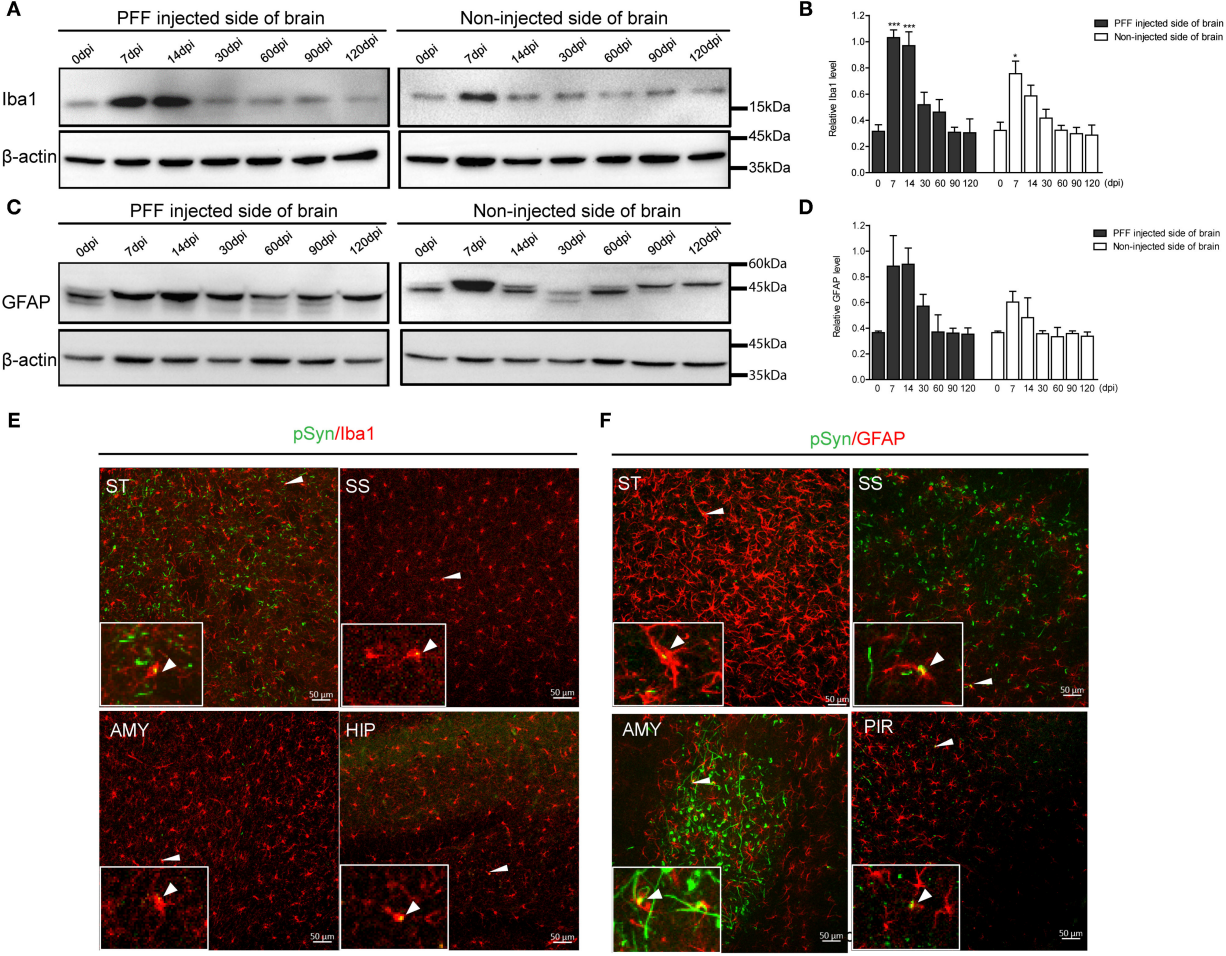
The change of Iba1 and GFAP expression in the whole brain homogenate and the relationship of pSyn and inflammatory cells. **(A,B)** A representative immunoblot image **(A)** and its quantification **(B)** showing the level of Iba1 in the PFF-injected side of the brain and the noninjected side of the brain at each time point. Relative Iba1 level in the whole brain went up and then down significantly (χ^2^ = 69.63, p < 0.0001, df = 13). **(C,D)** Immunoblot **(C)** and quantification data **(D)** showed the level of GFAP in the PFF-injected and noninjected side of the brain increased and then decreased (χ^2^ = 16.47, p = 0.2246, df = 13). **(E,F)** Spatial relationship of inflammatory cells—microglia **(E)** and astrocytes **(F)**—and pSyn aggregation in various brain regions. Confocal images showing double immunostaining of pSyn (green) and Iba1 or GFAP (red) in PFF-injected mice. White arrows indicate the colocalization of pSyn and microglia or astrocytes. ST, SS, AMY, HIP, and PIR are striatum, somatosensory cortex, amygdala, hippocampus, and piriform cortex, respectively. Original blots are presented in Supplementary Figure 7. Bars indicate SEM, n = 4–5/time point. Statistical significance was measured with a comparison of 0 dpi and each time point by a *post hoc* analysis (the Kruskal–Wallis test, ^*^p < 0.05, ^***^p < 0.001).

In a western blot analysis using the whole brain homogenate, microglia significantly increased at 7 dpi, and then decreased with time both in the noninjected and PFF-injected side of the brain (Figures 7A,B). A similar increasing trend in astrocytes was also observed but was not statistically significant as shown in Figures 7C,D.

In addition, some αSyn aggregations were observed inside microglia and astrocytes in various brain regions, such as the striatum, somatosensory cortex, amygdala, hippocampus, and piriform cortex, as shown in Figures 7E,F (see also Supplementary Figures 6A,B).

### Evaluation of the Behavioral Phenotype in the PD Mouse Model

**Figure 8:**
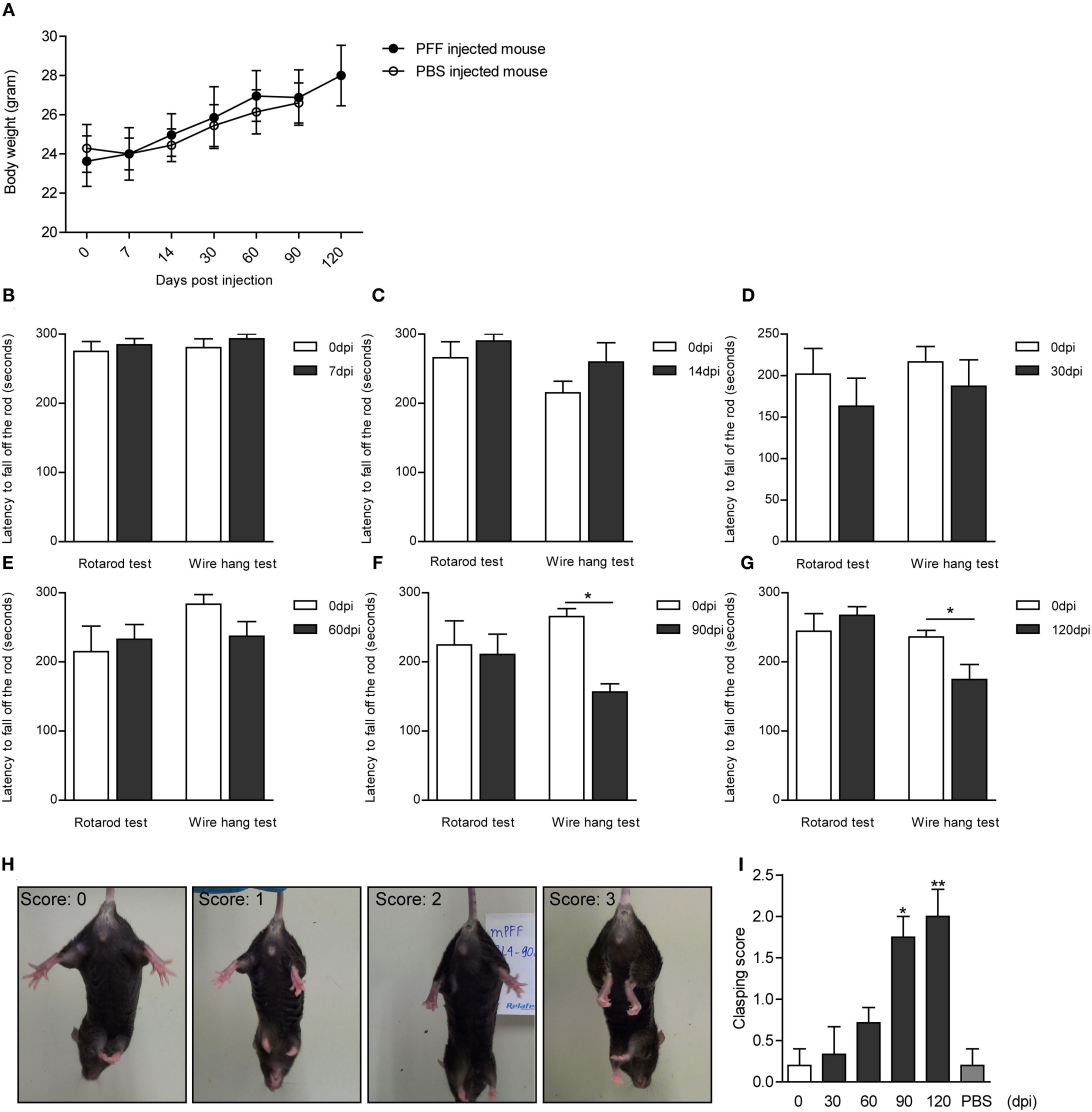
Body weight and behavioral changes in PFF-injected mouse. **(A)** Time-dependent changes in body weight did not show the difference between PFF-injected mice and PBS-injected mice (p > 0.05). **(B–G)** The results of the rotarod and wire hang test at each time point. The wire hang test showed significant motor deficits from 90 days, whereas the rotarod test did not show. The results were obtained from the same mice group, respectively, at baseline and at the time of sacrifice. Six to eight mice per group (mice with the same dpi at sacrifice) were used for each experiment. **(H,I)** Hind limb clasping test. **(H)** Representative images of mice exhibiting the different scores of hind limb clasping. **(I)** Clasping score increased significantly with time compared to baseline. For the clasping score analysis, six to eight mice were used at each time point for this analysis. For the PBS group, five mice were used at 90 dpi. Data are presented as mean ± SEM (Wilcoxon signed-rank test, ^*^p < 0.05, ^**^p < 0.01).

The serial bodyweight of mice at each time point until the sacrifice was recorded. The body weight was observed to increase over time for 120 days but did not show any difference between PFF- and PBS-injected mice as shown in Figure 8A (*p* > 0.05). To assess the manifestation of motor symptoms, latency to drop on rotarod and wire hang was measured at two-time points—at baseline (before injection) and sacrifice day as shown in Figures 8B–G. The time to drop on rotarod did not decrease at all time points compared to baseline while the wire hang test showed poor performance significantly at 90 (*p* = 0.031) and 120 dpi (*p* = 0.0195) compared to baseline. Hind limb clasping sign increased with time and increased significantly at 90 and 120 dpi (Figures 8H,I) (see also Supplementary Data 1).

### Temporal Evolution of Neuroinflammation, αSyn Propagation, and Neurodegeneration

**Figure 9:**
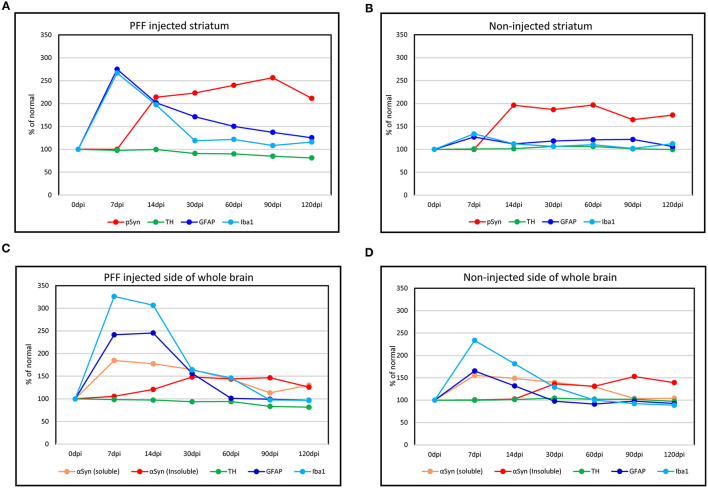
The temporal evolution of inflammation and neurodegeneration with αSyn propagation in the PFF-injected Parkinson's disease (PD) mouse model. **(A,B)** Line graphs showing the temporal relationship of inflammation (microglia by Iba1 and astrocytes by GFAP expression) and neurodegeneration (dopaminergic neuron by TH expression) with αSyn propagation (pSyn expression) in the striatum in the PFF-injected side **(A)** and the noninjected side **(B)**. **(C,D)** The temporal relationship of inflammation and dopaminergic neurodegeneration with the change of αSyn contents (αSyn soluble and αSyn insoluble expression) in the whole brain: the PFF-injected side brain **(C)** and noninjected side brain **(D)**.

Considering all predescribed study results, graphs were made to show the temporal relationship of neuroinflammation and neurodegeneration with αSyn propagation in the PFF-injected PD mouse model that is divided into a regional change in the striatum and a gross change in the whole brain. In the striatum, the neuroinflammation by microglia and astrocytes reached a peak at 7 dpi and then the peak tended to reduce with time while pSyn aggregation increased rapidly from 7 to 14 dpi and then the increasing rate settled down in the PFF-injected striatum. The loss of striatal dopaminergic neurons was noted to progress slowly until it became significant at 90 dpi in the PFF-injected striatum (Figure 9A). Furthermore, the noninjected striatum showed a similar pattern of inflammation and αSyn accumulation like that of the PFF-injected striatum even though the neuronal loss was not observed yet (Figure 9B).

Overall trends in the whole brain showed that inflammatory response cells, especially microglia, were considerably increased at 7 dpi and tended to reduce with time in both PFF-injected and noninjected sides of the brain (Figures 9C,D). Soluble αSyn level rapidly increased at 7 dpi in both sides of the brain, and then tended to reduce with time. The insoluble form of αSyn increased after 14 dpi and peaked at 30–90 dpi in both PFF-injected and noninjected sides of the brain. The reduction of total TH-positive dopaminergic neurons showed a tendency to decrease in the PFF-injected side of the brain. Altogether, inflammation started first, which was followed by αSyn accumulation, and later on, neurodegeneration began.

## Discussion

This study demonstrated the temporal relationship of inflammatory responses, αSyn propagation, and neurodegeneration in the PFF-injected PD mouse model. Overall, inflammatory response cells—microglia and astrocytes—reached their peak at the earliest stage, the increment of pathological αSyn accumulation followed behind, and then dopaminergic neurons started to degenerate with clinical phenotype manifestation.

This study suggested that PFF induced inflammation first—especially microglial activation—with increasing endogenous αSyn protein production and then its pathological accumulation in an order. This relationship was observed in both PFF-injected and noninjected striatums. Additionally, considering the PBS-injected mouse model and monomeric αSyn-injected mouse model, this early inflammatory activation is not only caused due to needle injury and surgical effect but also by a direct or an indirect effect of the introduction of PFF. Considering that monomeric αSyn induced less inflammation than αSyn PFF, which implied that inflammation is not only caused by the effect of endotoxin. One study by Duffy et al. (2018) reported the temporal change of αSyn inclusion formation, MHC-II expression, and neurodegeneration in the striatum from 1 to 6 months in the PFF-injected rat. Within the striatum, this study determined that MHC-II immunoreactivity increased after PFF injection and then decreased over time before the accumulation of αSyn. Moreover, a recent postmortem study evaluating PD brain receiving fetal nigral cell transplantation showed that microglial activation was observed with or even preceded αSyn accumulation in the grafted cells (Olanow et al., 2019). These studies suggested that inflammatory responses can start previously than pathological αSyn accumulation, which is similar to our study observations. In contrast to a previous thought that inflammation is the resultant findings of synucleinopathy, which might suggest that inflammation can precede or contribute to αSyn propagation.

Our study has evaluated the two specific types of CNS inflammatory response cells, i.e., astrocytes and microglia, in a mouse model. The PD mouse model in this study has shown that both astrocytes and microglia exhibited similar patterns—early high activation and following the decreasing pattern with time before the acceleration of pSyn accumulation. Particularly, the whole brain homogenate analysis revealed that microglia showed prominent activation in the bilateral brain irrespective of the PFF injection side while astrocytic activation was not significant statistically. One possible explanation is that microglial immune response has been determined to be more closely associated with the primary defense mechanism to the invasion of pathological αSyn-like PFF. Our previous study demonstrated that microglia activated preferentially to αSyn propagation compared to astroglia in the striatum at 1 month after PFF injection (Kim et al., 2020). Additionally, Lee et al. (2014) have reported that the activated microglia induce an A1 neuroinflammatory-reactive astrocyte, which is a neurotoxic phenotype rapidly killing neurons. As a result, microglia may react predominantly to pathological αSyn intrusion and might determine the fate of astrocytes using the next step.

This study observed that some microglia or astrocytes contained αSyn inclusions in different brain regions at various time points. These findings postulated that microglia or astrocytes may internalize the pathologic form of αSyn, for antigen presentation or possibly to clear it because of their role like that of a macrophage (Lee et al., 2014; Hickman et al., 2018; Choi et al., 2020). Guo et al. (2020) have also reported that microglial exosomes contribute to the progression of αSyn pathology, which was consistent with the finding of Xia et al. (2019) that microglia play a role in the transmission of αSyn *via* exosomal protein transfer. This may imply that increased or activated microglia may engulf pathologic αSyn protein to clear it up, but the fragmented αSyn was released by exosomes, which could be transferred to other neurons or cells and consequently enabled acceleration of the αSyn interneural transmission. αSyn-containing astrocytes were observed in the postmortem PD brain, which was found to be broader than Lewy body distribution (Braak et al., 2007). Sorrentino et al. (2017) described αSyn aggregates preferentially colocalized within astrocytes in human αSyn transgenic mice injected with human αSyn PFF. In addition to the macrophage-like action of astrocytes and microglia, another explanation regarding intracellular αSyn inclusions in these immune cells is that not only αSyn does transfer *via* interneuronal connection but also glia-neuronal transmission is involved in αSyn propagation. Further experiments regarding the association of microglia and astrocytes with αSyn propagation are thus necessary to understand the contribution of microglia and astrocytes during the disease process.

The loss of dopaminergic nerve fibers in the striatum from 90 dpi and of SN from 60 dpi was observed in the PFF-injected side while noninjected SN showed a trend of the loss of dopaminergic nerve fibers without statistical significance. Moreover, the noninjected striatum did not exhibit the dopaminergic neurodegeneration yet during the observation period. It implied that neurodegeneration occurs late or tardy because the noninjected striatum was affected by toxic αSyn indirectly or a smaller amount of αSyn than the PFF-injected striatum. At this moment, the neurodegeneration starts, which could be different from the study conditions while it is deemed consistent that the dopaminergic neurodegeneration followed by the accumulation of αSyn. Luk et al. (2012a) showed that the reduction of TH signal in the striatum following a unilateral PFF injection was observed at 180 days despite the occurrence of αSyn accumulation at 30 days. Patterson et al. (2019) have also noted that TH immunoreactivity in the striatum of a rat model seemed to be maintained or upregulated up to 2 months depending on the amount of injected PFF, which was followed by severe loss in 4 months, while αSyn accumulation peaked in 2 months. A study by Duffy et al. (2018) showed that αSyn accumulation was increased from 1 month after PFF injection, and this mouse model did not show any changes in TH-positive dopaminergic neurons in the striatum until 6 months while SN showed the loss of dopaminergic neuron by 5–6 months postinjection. Taking these findings into consideration, the loss of striatal dopaminergic nerve fibers occurred later after pathological αSyn accumulation became prominent. The observation in this study is in line with previous findings of the occurrence of dopaminergic degeneration in the striatum or SN following αSyn aggregation (Luk et al., 2012a; Karampetsou et al., 2017; Duffy et al., 2018; Patterson et al., 2019). Further experiments are thus needed to explore the mechanism underlying the loss of dopaminergic neurons like the accumulation of αSyn.

In this study, the accumulation and spread of αSyn were found in the whole brain, irrespective of the PFF injection side from the earliest time point of 7 days while Okuzumi et al. (2018) found early αSyn inclusions at 3 weeks postinjection. A few previous studies often reported αSyn accumulation at 1 month or longer through intrastriatal or intracerebral PFF injection into the mouse or rat (Luk et al., 2012a,b; Paumier et al., 2015; Patterson et al., 2019). Our data suggested that αSyn accumulation was formed very fast after PFF injection, which was the earliest observation compared to previous studies (Luk et al., 2012a,b; Paumier et al., 2015; Patterson et al., 2019).

Additionally, the changing shapes of the aggregated αSyn inclusions in various brain regions have been observed over time. With time, the shapes of the αSyn inclusions changed or matured from dot- or thread-like (Lewy neurite-like shape) structures to cytoplasmic ring-like structures (Lewy body-like shape). Moreover, one interesting finding is that the piriform cortex and amygdala showed the matured shape of αSyn accumulation, i.e., Lewy body-like structure, earlier than the other brain regions, including also the striatum. This finding suggested that pathological αSyn propagated rapidly from the striatum to other brain regions, and different brain regions showed respective speeds of αSyn maturation but was not in accordance with the distance from the PFF injection site. Okuzumi et al. (2018) have also reported the change in the shape of aggregated αSyn with time. Further study on the morphological maturation of αSyn accumulation is needed to evaluate whether the difference of regional maturation speed is determined by regional neuronal characteristics and whether these different morphologies of αSyn have respective neuronal toxicities.

As disease progresses, αSyn undergoes conformational changes to form oligomers and high molecular weight aggregates, including the phosphorylated form of αSyn that tend to make the protein more insoluble (Bandopadhyay, 2016). Therefore, we applied a methodology to describe the alteration in the content of soluble αSyn and insoluble αSyn in our PD mouse model. Interestingly, this study found that the level of soluble αSyn rapidly increased at an early time after PFF injection and then tended to reduce at a later time in both sides of the brain irrespective of PFF injection. After soluble αSyn increased, the insoluble αSyn or pSyn increased in NP-40 insoluble fraction in the brain. Moreover, a band with the lower molecular weight than full-length αSyn bands of 15 kDa was observed in the insoluble fraction and was not detectable in the soluble fraction as shown in Figures 4A,C. It possibly indicates a C-terminal-truncated αSyn form of αSyn. Nuber et al. (2013) previously described that aging promoted the conversion of both full-length and C-terminally truncated αSyn species, which were detected into the insoluble fraction in the striatum of human bacterial artificial chromosome (BAC) αSyn transgenic rat. Likewise, Games et al. (2013) reported that the accumulation of C-truncated αSyn was detectable using the western blotting of mice overexpressing human αSyn at 6 months. Altogether, our data have collectively suggested that endogenous αSyn expression started to increase as soon as PFF was injected, and then increasing αSyn contents changed to an insoluble form or a phosphorylated form and later enabling their accumulation in an intracellular area.

Regarding the motor phenotype during the αSyn aggregation, the wire hang test and clasping sign were recently found to be more sensitive in detecting the movement phenotype than the rotarod test in this mouse model. These two tests displayed a significant motor impairment from 90 days after injection. From the previous references, the manifestation of the motor phenotype was reported to vary depending on the test methods and study designs used. Luk et al. (2012a) reported that no impairment was observed in rotarod test up to 180 days after intrastriatal PFF injection even though the poor performance was detected on the wire hang test at an earlier time, declining 53% from baseline at 30 dpi and 81% at 180 dpi, which was concomitant with the dopaminergic neuron loss in SN by 15 and 35% at 90 and 180 dpi, respectively. Yun et al. (2018) reported the reduction of falling time at 6 months after intrastriatal PFF injection compared to PBS injection by the rotarod test, and PFF injection in mice induced a significant loss of TH and Nissl-positive neurons in SN while Karampetsou et al. (2017) did not observe a significant difference in the rotarod test, pole test, and the grip strength in PFF-injected mice at 60 dpi, which showed a significant reduction of dopaminergic neuron. Additionally, Stoyka et al. (2020) reported the lack of motor deficits in mice, which received bilateral injection of αSyn fibrils at 6 months, especially no significant difference in latency to fall on accelerating rotarod testing while 18% loss of NeuN-positive neuron was observed in the amygdala. For the lipopolysaccharide (LPS) administration in the Thy1-αSyn mouse model, Gorecki et al. (2019) reported a significantly increased mean hind limb clasp reflex score compared to baseline. Our study found that motor deficit showed prominently at the time the significance of dopaminergic neurodegeneration. Importantly, these observations suggested that neurodegeneration, rather than αSyn aggregation, might seem to be accompanied by motor behavioral abnormalities, and our data support other studies showing the motor impairments in various rodent models of PD (Luk et al., 2012a; Sharma and Nehru, 2015; Wrangel et al., 2015; Edwards et al., 2019; Kim et al., 2019). Overall, these behavioral assessments allowed the recognition of the PD-like motor symptoms in PFF-injected mice, but utilizing further behavioral assessment methods might be necessary to also investigate the nonmotor dysfunction phenotype in the αSyn mouse model.

To exclude the endotoxin effect, we compared the inflammation by αSyn monomer and αSyn PFF injection and showed that the inflammation was more prominent in αSyn PFF-injected brain compared with αSyn monomer-injected brain by image. However, this result is limited in the case of not quantifying the inflammation between the two groups.

In this study, we are interested in the temporal relationship of neuroinflammation and neurodegeneration with αSyn propagation in the PFF-injected PD mouse model. Although this study only focused on the temporal change of the striatum and the whole brain, the evaluation for the other brain regions will be informative as well. In summary, our findings support that in the PD mouse model, the peak of neuroinflammation occurred first, followed by αSyn accumulation, and, later on, neurodegeneration with behavioral phenotype. Further investigations regarding the causal relationship of inflammation, αSyn propagation, and neurodegeneration are thus necessary.

## Data Availability Statement

The original contributions presented in the study are included in the article/Supplementary Material, further inquiries can be directed to the corresponding author.

## Ethics Statement

The animal study was reviewed and approved by The Animal Research Committee of Hallym University Sacred Heart Hospital.

## Author Contributions

TL designed and performed all the experiments, analyzed data, prepared the figures, and wrote the manuscript. YK conceived and designed experiments and reviewed the manuscript. PN and YK performed recombinant αSyn. TN took apart in tissue sections collection. H-iM conceived and designed experiments and reviewed the manuscript. YK conceived and supervised the work, prepared the figures, and wrote the manuscript. All authors read and approved the final manuscript.

## Funding

This research was supported by Hallym University Research Fund and the National Research Foundation of Korea (NRF) grant funded by the Korean government (MSIT) (2017R1C1B5076402 and 2020R1F1A1076697).

## Conflict of Interest

The authors declare that the research was conducted in the absence of any commercial or financial relationships that could be construed as a potential conflict of interest.

## Publisher's Note

All claims expressed in this article are solely those of the authors and do not necessarily represent those of their affiliated organizations, or those of the publisher, the editors and the reviewers. Any product that may be evaluated in this article, or claim that may be made by its manufacturer, is not guaranteed or endorsed by the publisher.

## References

[B1] Bandopadhyay R. (2016). Sequential extraction of soluble and insoluble alpha-synuclein from parkinsonian brains. J. Vis. Exp. 2016:53415. 10.3791/53415 26780369 PMC4781043

[B2] Braak H. Sastre M. Del Tredici K. (2007). Development of α-synuclein immunoreactive astrocytes in the forebrain parallels stages of intraneuronal pathology in sporadic Parkinson's disease. Acta Neuropathol. 114, 231–241. 10.1007/s00401-007-0244-3 17576580

[B3] Brás I. C. Dominguez-Meijide A. Gerhardt E. Koss D. Lázaro D. F. Santos P. I.. (2020). Synucleinopathies: where we are and where we need to go. J. Neurochem. 153, 433–454. 10.1111/jnc.14965 31957016

[B4] Choi I. Zhang Y. Seegobin S. P. Pruvost M. Wang Q. Purtell K.. (2020). Microglia clear neuron-released α-synuclein via selective autophagy and prevent neurodegeneration. Nat. Commun. 11, 1–14. 10.1038/s41467-020-15119-w 32170061 PMC7069981

[B5] Chung H. K. Ho H.-A. Pérez-Acuña D. Lee S.-J. (2019). Modeling α-synuclein propagation with preformed fibril injections. J. Mov. Disord. 12, 139–151. 10.14802/jmd.19046 31556259 PMC6763716

[B6] Crowe A. Yue W. (2019). Semi-quantitative determination of protein expression using immunohistochemistry staining and analysis: an integrated protocol. Bio-Protocol 9:e3465. 10.21769/bioprotoc.3465 31867411 PMC6924920

[B7] Duffy M. F. Collier T. J. Patterson J. R. Kemp C. J. Luk K. C. Tansey M. G.. (2018). Lewy body-like alpha-synuclein inclusions trigger reactive microgliosis prior to nigral degeneration. J. Neuroinflam. 15:129. 10.1186/s12974-018-1171-z 29716614 PMC5930695

[B8] Edwards G. Gamez N. Armijo E. Kramm C. Morales R. Taylor-Presse K.. (2019). Peripheral delivery of neural precursor cells ameliorates parkinson's disease-associated pathology. Cells 8:1359. 10.3390/cells8111359 31671704 PMC6912680

[B9] Games D. Seubert P. Rockenstein E. Patrick C. Trejo M. Ubhi K.. (2013). Axonopathy in an α-synuclein transgenic model of Lewy body disease is associated with extensive accumulation of c-terminal-truncated α-synuclein. Am. J. Pathol. 182, 940–953. 10.1016/j.ajpath.2012.11.018 23313024 PMC3589076

[B10] Gorecki A. M. Preskey L. Bakeberg M. C. Kenna J. E. Gildenhuys C. MacDougall G.. (2019). Altered gut microbiome in Parkinson's disease and the influence of lipopolysaccharide in a human α-synuclein over-expressing mouse model. Front. Neurosci. 13:839. 10.3389/fnins.2019.00839 31440136 PMC6693556

[B11] Guo M. Wang J. Zhao Y. Feng Y. Han S. Dong Q.. (2020). Microglial exosomes facilitate a-synuclein transmission in Parkinson's disease. Brain 143, 1476–1497. 10.1093/brain/awaa090 32355963 PMC7241957

[B12] Harms A. S. Kordower J. H. Sette A. Lindestam Arlehamn C. S. Sulzer D. Mach R. H. (2020). Inflammation in experimental models of α-synucleinopathies. Mov. Disord. 36, 37–49. 10.1002/mds.28264 33009855 PMC8115204

[B13] Hickman S. Izzy S. Sen P. Morsett L. El Khoury J. (2018). Microglia in neurodegeneration. Nat. Neurosci. 21, 1359–1369. 10.1038/s41593-018-0242-x 30258234 PMC6817969

[B14] Hirsch E. C. Standaert D. G. (2020). Ten unsolved questions about neuroinflammation in Parkinson's disease. Mov. Disord. 36, 16–24. 10.1002/mds.28075 32357266

[B15] Karampetsou M. Ardah M. T. Semitekolou M. Polissidis A. Samiotaki M. Kalomoiri M.. (2017). Phosphorylated exogenous alpha-synuclein fibrils exacerbate pathology and induce neuronal dysfunction in mice. Sci. Rep. 7:16533. 10.1038/s41598-017-15813-8 29184069 PMC5705684

[B16] Kim S. Kwon S. H. Kam T. I. Panicker N. Karuppagounder S. S. Lee S.. (2019). Transneuronal propagation of pathologic α-synuclein from the gut to the brain models Parkinson's disease. Neuron 103, 627–641.e7. 10.1016/j.neuron.2019.05.035 31255487 PMC6706297

[B17] Kim Y. E. Lai T. T. Kim Y. J. Jeon B. (2020). Preferential microglial activation associated with pathological alpha synuclein transmission. J. Clin. Neurosci. 81, 469–476. 10.1016/j.jocn.2020.09.027 33222964

[B18] Kouli A. Torsney K. M. Kuan W.-L. (2018). Parkinson's Disease: Etiology, Neuropathology, and Pathogenesis, in Parkinson's Disease: Pathogenesis and Clinical Aspects (Brisbane: Exon Publications), 3–26. 10.15586/codonpublications.parkinsonsdisease.2018.ch1

[B19] Lee H. J. Bae E. J. Lee S. J. (2014). Extracellular α-synuclein-a novel and crucial factor in Lewy body diseases. Nat. Rev. Neurol. 10, 92–98. 10.1038/nrneurol.2013.275 24468877

[B20] Luk K. C. Kehm V. Carroll J. Zhang B. O'Brien P. Trojanowski J. Q.. (2012a). Pathological α-synuclein transmission initiates Parkinson-like neurodegeneration in nontransgenic mice. Science 338, 949–953. 10.1126/science.1227157 23161999 PMC3552321

[B21] Luk K. C. Kehm V. M. Zhang B. O'Brien P. Trojanowski J. Q. Lee V. M. Y. (2012b). Intracerebral inoculation of pathological α-synuclein initiates a rapidly progressive neurodegenerative α-synucleinopathy in mice. J. Exp. Med. 209, 975–988. 10.1084/jem.20112457 22508839 PMC3348112

[B22] Miedel C. J. Patton J. M. Miedel A. N. Miedel E. S. Levenson J. M. (2017). Assessment of spontaneous alternation, novel object recognition and limb clasping in transgenic mouse models of amyloid-β and tau neuropathology. J. Vis. Exp. 2017:55523. 10.3791/55523 28605382 PMC5608159

[B23] Nuber S. Harmuth F. Kohl Z. Adame A. Trejo M. Schönig K.. (2013). A progressive dopaminergic phenotype associated with neurotoxic conversion of α-synuclein in BAC-transgenic rats. Brain 136, 412–432. 10.1093/brain/aws358 23413261 PMC3572936

[B24] Okuzumi A. Kurosawa M. Hatano T. Takanashi M. Nojiri S. Fukuhara T.. (2018). Rapid dissemination of alpha-synuclein seeds through neural circuits in an in-vivo prion-like seeding experiment. Acta Neuropathol. Commun. 6:96. 10.1186/s40478-018-0587-0 30231908 PMC6145187

[B25] Olanow C. W. Savolainen M. Chu Y. Halliday G. M. Kordower J. H. (2019). Temporal evolution of microglia and α-synuclein accumulation following foetal grafting in Parkinson's disease. Brain 142, 1690–1700. 10.1093/brain/awz104 31056668

[B26] Patterson J. R. Duffy M. F. Kemp C. J. Howe J. W. Collier T. J. Stoll A. C.. (2019). Time course and magnitude of alpha-synuclein inclusion formation and nigrostriatal degeneration in the rat model of synucleinopathy triggered by intrastriatal α-synuclein preformed fibrils. Neurobiol. Dis. 130:104525. 10.1016/j.nbd.2019.104525 31276792 PMC6701176

[B27] Paumier K. L. Luk K. C. Manfredsson F. P. Kanaan N. M. Lipton J. W. Collier T. J.. (2015). Intrastriatal injection of pre-formed mouse α-synuclein fibrils into rats triggers α-synuclein pathology and bilateral nigrostriatal degeneration. Neurobiol. Dis. 82, 185–199. 10.1016/j.nbd.2015.06.003 26093169 PMC4640952

[B28] Rostami J. Fotaki G. Sirois J. Mzezewa R. Bergström J. Essand M.. (2020). Astrocytes have the capacity to act as antigen-presenting cells in the Parkinson's disease brain. J. Neuroinflammation 17:119. 10.1186/s12974-020-01776-7 32299492 PMC7164247

[B29] Samii A. Nutt J. G. Ransom B. R. (2004). Parkinson's disease. Lancet 2004, 1783–1793. 10.1016/S0140-6736(04)16305-8 15172778

[B30] Sasaki A. Arawaka S. Sato H. Kato T. (2015). Sensitive western blotting for detection of endogenous Ser129-phosphorylated α-synuclein in intracellular and extracellular spaces. Sci. Rep. 5, 1–15. 10.1038/srep14211 26381815 PMC4585644

[B31] Sharma N. Nehru B. (2015). Characterization of the lipopolysaccharide induced model of Parkinson's disease: role of oxidative stress and neuroinflammation. Neurochem. Int. 87, 92–105. 10.1016/j.neuint.2015.06.004 26055970

[B32] Sorrentino Z. A. Brooks M. M. T. Hudson V. Rutherford N. J. Golde T. E. Giasson B. I.. (2017). Intrastriatal injection of α-synuclein can lead to widespread synucleinopathy independent of neuroanatomic connectivity. Mol. Neurodegener. 12, 1–16. 10.1186/s13024-017-0182-z 28552073 PMC5447308

[B33] Stefanova N. Georgievska B. Eriksson H. Poewe W. Wenning G. K. (2012). Myeloperoxidase inhibition ameliorates multiple system atrophy-like degeneration in a transgenic mouse model. Neurotox. Res. 21, 393–404. 10.1007/s12640-011-9294-3 22161470

[B34] Stoyka L. E. Arrant A. E. Thrasher D. R. Russell D. L. Freire J. Mahoney C. L.. (2020). Behavioral defects associated with amygdala and cortical dysfunction in mice with seeded α-synuclein inclusions. Neurobiol. Dis. 134:104708. 10.1016/j.nbd.2019.104708 31837424 PMC7206936

[B35] Tagliaferro P. Ramos A. J. Onaivi E. S. Evrard S. G. Vega M. D. Brusco A. (2006). Morphometric study on cytoskeletal components of neuronal and astroglial cells after chronic CB1 agonist treatment. Methods Mol. Med. 123, 91–104. 10.1385/1-59259-999-0:91 16506403

[B36] Wrangel C. von Schwabe K. John N. Krauss J. K. Alam M. (2015). The rotenone-induced rat model of Parkinson's disease: behavioral and electrophysiological findings. Behav. Brain Res. 279, 52–61. 10.1016/j.bbr.2014.11.002 25446762

[B37] Xia Y. Zhang G. Han C. Ma K. Guo X. Wan F.. (2019). Microglia as modulators of exosomal alpha-synuclein transmission. Cell Death Dis. 10, 1–15. 10.1038/s41419-019-1404-9 30787269 PMC6382842

[B38] Yun S. P. Kam T. I. Panicker N. Kim S. Oh Y. Park J. S.. (2018). Block of A1 astrocyte conversion by microglia is neuroprotective in models of Parkinson's disease. Nat. Med. 24, 931–938. 10.1038/s41591-018-0051-5 29892066 PMC6039259

