# Supplementary material for: Temporal Evolution of Inflammation and Neurodegeneration With Alpha-Synuclein Propagation in Parkinson's Disease Mouse Model
Source: Front Integr Neurosci. 2021 Oct 5;15:715190. doi: 10.3389/fnint.2021.715190 (PMC8523784; doi:10.3389/fnint.2021.715190)
Supplement: Supplementary file 1 [file Image_1.PDF]

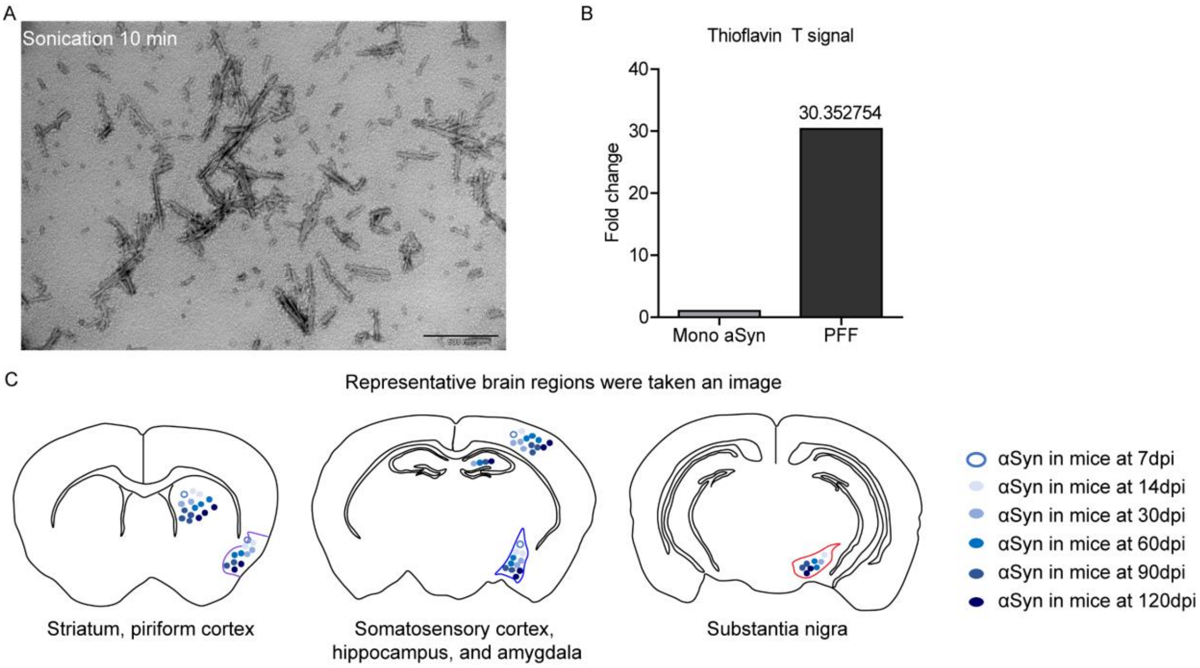

Supplementary Figure 1: Quality assessment of the αSyn preformed fibrils (A) Preformed fibrils by transmission electron microscopy (TEM), scale of 500 nm and **(B)** Thioflavin T (ThT, Sigma, T3516) assay were conducted to verify fibril formation. **(C)** Representative time-dependent pathological αSyn distribution in different brain regions used in this study. Abbreviation: dpi, day post injection.
