# Supplementary material for: Temporal Evolution of Inflammation and Neurodegeneration With Alpha-Synuclein Propagation in Parkinson's Disease Mouse Model
Source: Front Integr Neurosci. 2021 Oct 5;15:715190. doi: 10.3389/fnint.2021.715190 (PMC8523784; doi:10.3389/fnint.2021.715190)
Supplement: Supplementary file 2 [file Image_2.PDF]

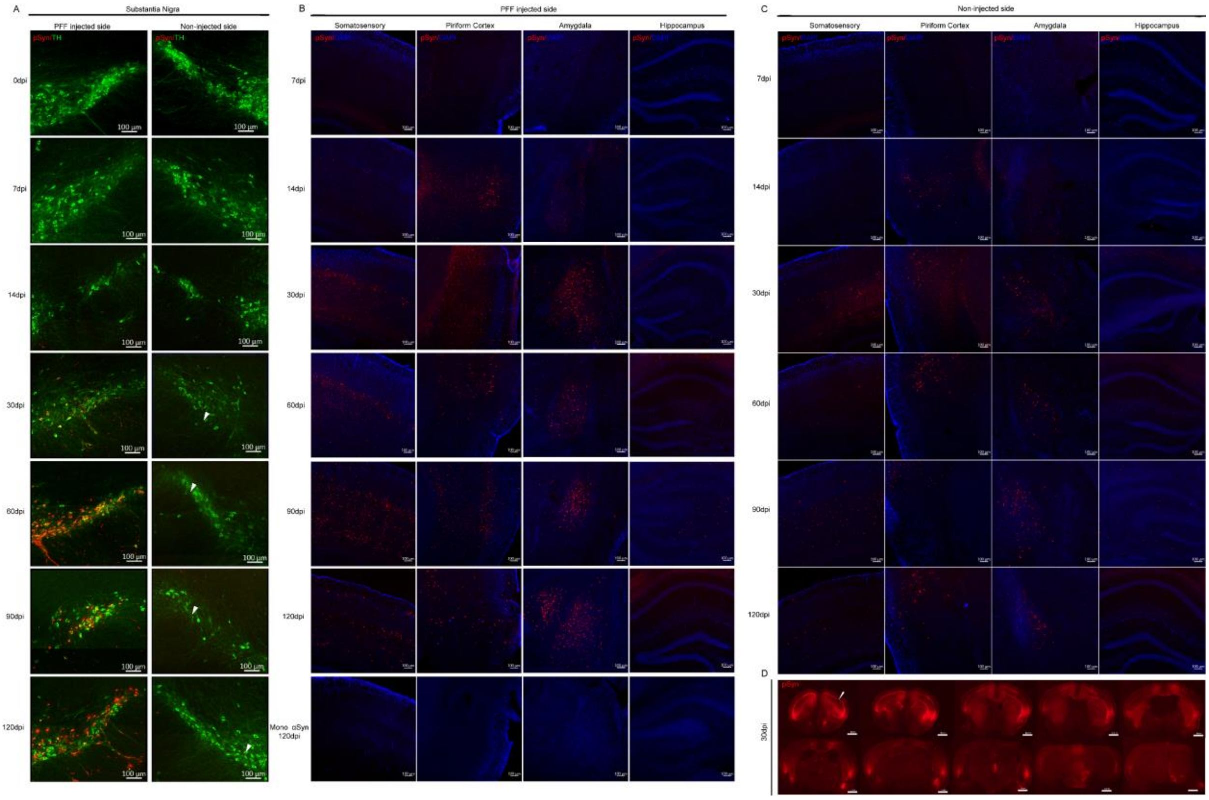

Supplementary Figure 2: The presence of pathological αSyn in various brain regions (A) Representative image of pSyn inclusions in SN at each time point. Double immunofluorescence staining with pSyn (red) and TH-positive dopaminergic neurons (green) in SN of PFF injected side (left column) and SN of non-injected side (right column). pSyn positive inclusions inside the dopaminergic neurons was observed prominently with time in SN, especially more in the PFF injected side. White arrow indicates the pSyn inclusion in SN of non-injected side. **(B, C)** Representative images of pSyn inclusions in different brain areas at each time point in PFF injected side **(B)** and non-injected side **(C)**. Immunofluorescence analysis of pSyn (red) with DAPI (blue) was performed in somatosensory cortex, piriform cortex, amygdala, and hippocampus of PFF-injected mice or αSyn monomer-injected mouse (mono αSyn) at different inoculation time. **(D)** Serial brain sections stained with pSyn of PFF injected mouse at 30dpi showing the wide pathological αSyn inclusions in various brain regions, white arrow indicates PFF injection side. Scale bar, 100 µm and 1mm.
