# Supplementary material for: Temporal Evolution of Inflammation and Neurodegeneration With Alpha-Synuclein Propagation in Parkinson's Disease Mouse Model
Source: Front Integr Neurosci. 2021 Oct 5;15:715190. doi: 10.3389/fnint.2021.715190 (PMC8523784; doi:10.3389/fnint.2021.715190)
Supplement: Supplementary file 4 [file Image_4.PDF]

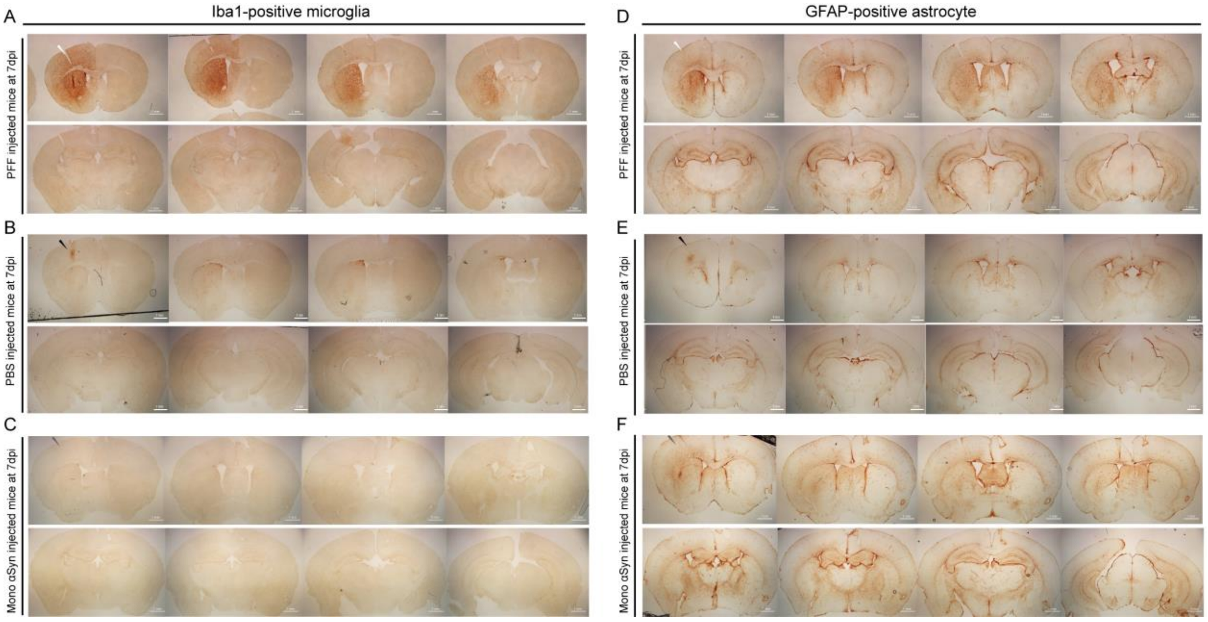

Supplementary Figure 4: Microglial and astrocytic activation in PFF injected mouse, PBS injected mouse and monomeric αSyn injected mouse at 7dpi (A) Serial brain sections stained with Iba1 antibody of PFF injected mouse showing abundant Iba1-positive microglia immunoreactivity. **(B, C)** Serial brain sections of PBS injected mouse and monomeric αSyn injected mouse at 7dpi stained Iba1 antibody, respectively. **(D)** Serial brain sections stained GFAP antibody of PFF injected mouse at 7dpi showing abundant GFAP-positive astrocyte immunoreactivity. **(E, F)** Serial brain sections of PBS injected mouse and monomeric αSyn injected mouse stained with GFAP antibody, respectively. PFF injected side as marked with the white arrow, PBS injected side as marked with the black arrow, and monomeric αSyn injected side as marked with the grey arrow. Scale bar, 1 mm.
