# Supplementary material for: Temporal Evolution of Inflammation and Neurodegeneration With Alpha-Synuclein Propagation in Parkinson's Disease Mouse Model
Source: Front Integr Neurosci. 2021 Oct 5;15:715190. doi: 10.3389/fnint.2021.715190 (PMC8523784; doi:10.3389/fnint.2021.715190)
Supplement: Supplementary file 5 [file Image_5.PDF]

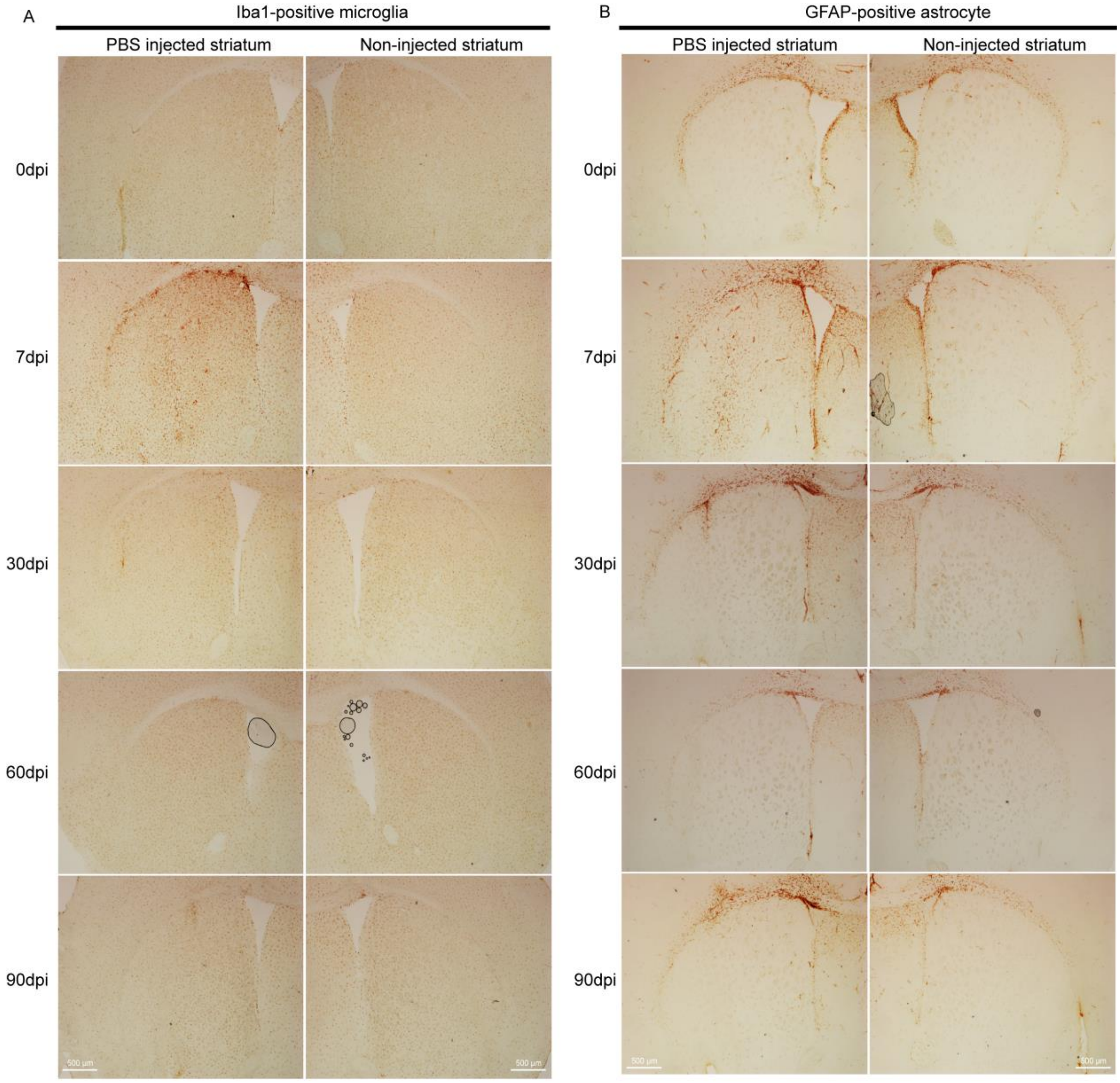

Supplementary Figure 5: Temporal change of microglial and astrocytic activation in PBS injected mouse (A, B) DAB staining showed microglia and astrocyte expression in PBS injected mouse at different time post injection. Scale bar, 500 µm.
