# Supplementary material for: Temporal Evolution of Inflammation and Neurodegeneration With Alpha-Synuclein Propagation in Parkinson's Disease Mouse Model
Source: Front Integr Neurosci. 2021 Oct 5;15:715190. doi: 10.3389/fnint.2021.715190 (PMC8523784; doi:10.3389/fnint.2021.715190)
Supplement: Supplementary file 6 [file Image_6.PDF]

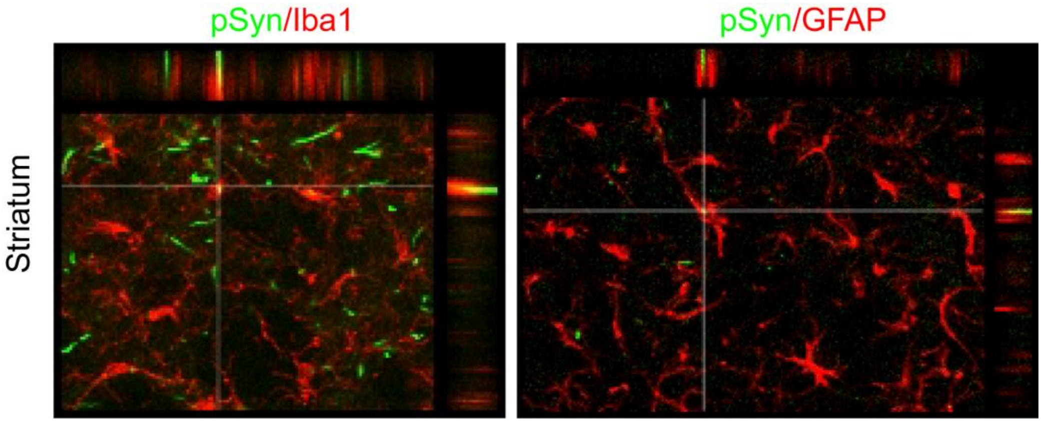

Supplementary Figure 6: Colocalization of pSyn (green) and Iba1 or GFAP (red) in the striatum of the PFF injected mice.
