# Supplementary material for: Temporal Evolution of Inflammation and Neurodegeneration With Alpha-Synuclein Propagation in Parkinson's Disease Mouse Model
Source: Front Integr Neurosci. 2021 Oct 5;15:715190. doi: 10.3389/fnint.2021.715190 (PMC8523784; doi:10.3389/fnint.2021.715190)
Supplement: Supplementary file 8 [file Table_1.DOCX]

**Supplementary Table 1: List of antibodies used for IF, IHC, and western blot**

| Antibodies | Source/ Cat | Working dilution |
| --- | --- | --- |
| Sheep Anti-Tyrosine hydroxylase | Abcam (ab113) | 1:1000 (IF) |
| Mouse Anti-Tyrosine hydroxylase | Immunostar (22941) | 1:1000 (IHC, WB) |
| Rabbit Anti-pSyn | Abcam (ab51253) | 1:1000 (IF, IHC, WB) |
| Rabbit Anti-αSyn | Abcam (ab212184) | 1:1000 (WB) |
| Goat Anti-Iba1 | Abcam (ab5076) | 1:1000 (IF) |
| Rabbit Anti-Iba1 | Wako (016-20001) | 1:1000 (IHC, WB) |
| Goat Anti-GFAP | Abcam (ab53554) | 1:500 (IF) |
| Mouse Anti-GFAP | Milipore (MAB360) | 1:1000 (WB), 1:500 (IHC) |
| Mouse Anti-β-actin | Santa cruz biotechnology (sc47778) | 1:1000 (WB) |
| Donkey Anti-Sheep IgG (AF488) | Abcam (ab150177) | 1:1000 (IF) |
| Donkey Anti-Rabbit IgG (AF555) | Abcam (ab150074) | 1:1000 (IF) |
| Donkey Anti-Rabbit IgG (DyLight488) | Bethyl laboratories.inc (A120-108D2) | 1:500 (IF) |
| Donkey Anti-Goat IgG (DyLight550) | Bethyl laboratories.inc (A50-101D3) | 1:500 (IF) |
| Goat Anti-Rabbit IgG (HRP conjugate) | Enzo (ADI-SAB-300-J) | 1:1000 (IHC), 1:5000 (WB) |
| Goat Anti-Mouse IgG (HRP conjugate) | Enzo (ADI-SAB-100-J) | 1:1000 (IHC), 1:5000 (WB) |
