# Supplementary material for: Temporal Evolution of Inflammation and Neurodegeneration With Alpha-Synuclein Propagation in Parkinson's Disease Mouse Model
Source: Front Integr Neurosci. 2021 Oct 5;15:715190. doi: 10.3389/fnint.2021.715190 (PMC8523784; doi:10.3389/fnint.2021.715190)
Supplement: Supplementary file 9 [file Data_Sheet_1.DOCX]

**Supplementary data 1:** **Clasping test of mouse at 0dpi, 90dpi, and 120dpi of PFF injection**

0dpi: <https://figshare.com/s/641c77e6fc2d3f85b862>

90dpi: <https://figshare.com/s/548e9b586ecb018301d6>

120dpi: <https://figshare.com/s/d20e248590a10df4d246>
